# A Genome-Wide Survey of Imprinted Genes in Rice Seeds Reveals Imprinting Primarily Occurs in the Endosperm

**DOI:** 10.1371/journal.pgen.1002125

**Published:** 2011-06-23

**Authors:** Ming Luo, Jennifer M. Taylor, Andrew Spriggs, Hongyu Zhang, Xianjun Wu, Scott Russell, Mohan Singh, Anna Koltunow

**Affiliations:** 1 CSIRO Plant Industry, Canberra, Australia; 2 Rice Research Institute of Sichuan Agricultural University, Chengdu, Sichuan, China; 3 Department of Botany and Microbiology, University of Oklahoma, Norman, Oklahoma, United States of America; 4 Plant Molecular Biology and Biotechnology Laboratory, The University of Melbourne, Melbourne, Australia; 5 CSIRO Plant Industry, Waite Campus, Adelaide, Australia; National Institute of Genetics, Japan

## Abstract

Genomic imprinting causes the expression of an allele depending on its parental origin. In plants, most imprinted genes have been identified in *Arabidopsis* endosperm, a transient structure consumed by the embryo during seed formation. We identified imprinted genes in rice seed where both the endosperm and embryo are present at seed maturity. RNA was extracted from embryos and endosperm of seeds obtained from reciprocal crosses between two subspecies Nipponbare (Japonica rice) and 93-11 (Indica rice). Sequenced reads from cDNA libraries were aligned to their respective parental genomes using single-nucleotide polymorphisms (SNPs). Reads across SNPs enabled derivation of parental expression bias ratios. A continuum of parental expression bias states was observed. Statistical analyses indicated 262 candidate imprinted loci in the endosperm and three in the embryo (168 genic and 97 non-genic). Fifty-six of the 67 loci investigated were confirmed to be imprinted in the seed. Imprinted loci are not clustered in the rice genome as found in mammals. All of these imprinted loci were expressed in the endosperm, and one of these was also imprinted in the embryo, confirming that in both rice and *Arabidopsis* imprinted expression is primarily confined to the endosperm. Some rice imprinted genes were also expressed in vegetative tissues, indicating that they have additional roles in plant growth. Comparison of candidate imprinted genes found in rice with imprinted candidate loci obtained from genome-wide surveys of imprinted genes in *Arabidopsis* to date shows a low degree of conservation, suggesting that imprinting has evolved independently in eudicots and monocots.

## Author Summary

The expression of maternal or paternal alleles in either a preferentially or exclusively uniparental manner, termed imprinting, is prevalent in the transient endosperm of seeds in the model plant *Arabidopsis*. Cereals form seeds where both the embryo and endosperm are present at seed maturity. They are an important world food source. To date, very few imprinted genes have been identified in cereal seeds. How parental gene expression biases contribute to rice seed development has not yet been studied in detail. The deep resolution of transcript sequencing platforms was used to identify loci expressed in a parentally biased manner in the embryo and endosperm of Indica and Japonica rice at a genome-wide level. We identified 262 candidate imprinted loci expressed in the endosperm, experimentally verified 56 of these, and found novel features pertaining to their expression. Only one gene was found to be imprinted in the rice embryo. Imprinting in *Arabidopsis* and rice seeds is confined primarily to the endosperm, but the identified loci do not share extensive sequence conservation. Imprinting thus appears to have evolved independently in these plant species.

## Introduction

Maternal and paternal alleles, inherited in plants and animals after fertilization, are usually equivalently expressed during the developmental cycle. In plants and in placental animals, a subset of genes is preferentially transcribed depending on the gender of the parent from which the gene originates and they are defined as imprinted genes [1]. Mono-allelic expression and also preferential expression of parental alleles has been observed for imprinted genes. A combination of epigenetic processes including DNA and histone methylation and demethylation are involved in repressing expression from one parental allele and enabling expression from the other in plants and animals [2]–[16].

**Figure 1:**
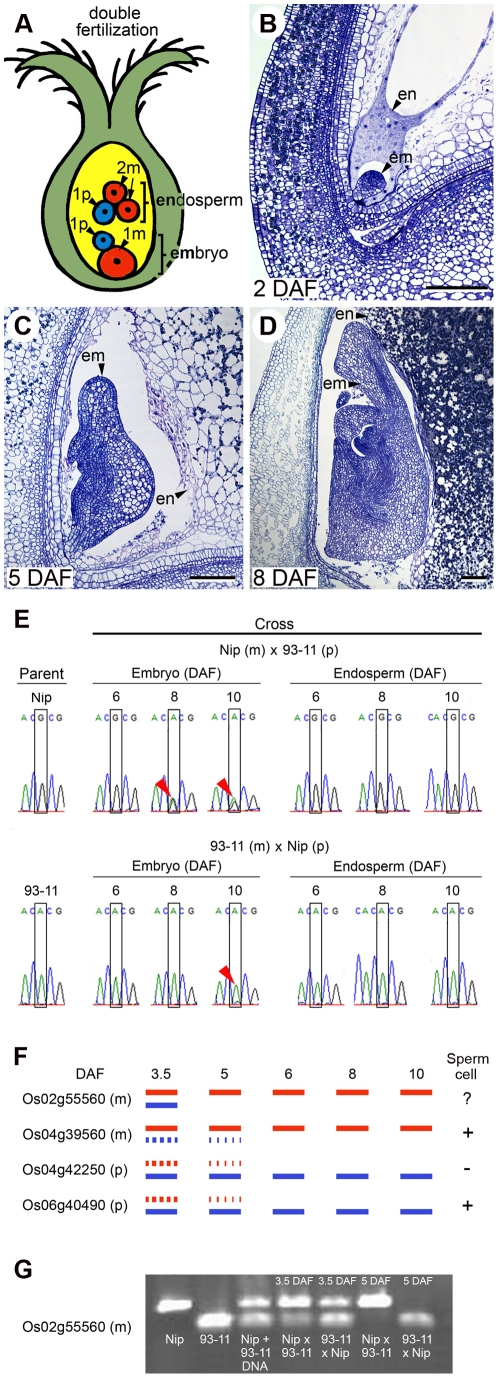
Seed development and imprinting in rice seeds. (A) A cartoon showing parental contributions to embryo and endosperm tissues following double fertilization in rice ovules. In the triploid endosperm the maternal (m) to paternal (p) genome ratio is 2∶1 and in the diploid embryo a 1m∶1p ratio is observed. (B–D) Sections of developing rice seeds at indicated days after fertilization (DAF) showing embryo (em) and endosperm (en) morphology and the proximity of these tissues. Embryo differentiation is complete 10 DAF. Starch and protein accumulation continue in the endosperm until grain maturation (∼20–25 DAF). Endosperm was harvested for transcriptome analyses at 5 DAF and embryos at 6 DAF. Scale bars are all 100 µm. (E) Os10g05750 is a maternally expressed gene found in the embryo and endosperm. RT-PCR sequencing chromatographs show that maternal expression is maintained during endosperm development. In the embryo imprinting is transient as bi-allelic expression is evident at 8 and 10 DAF in the Nip (m)×93-11 (p) cross (arrows indicate double peaks for G and A). Some paternal expression is evident in the embryo at 10 DAF in the 93-11 (m)×Nip (p) cross. Os10g05750 is expressed in a range of other plant tissues (Figure S6). (F) Temporal changes in imprinting status for four endosperm imprinted genes. The bars provide a schematic representation of the relative expression levels of maternal (m; red) or paternal (p; blue) alleles for the four genes based on RT-PCR sequencing chromatographs (Figure S3). Dotted bars indicate low level of expression. All four genes become uniparentally expressed. Expression in sperm cells is indicated by (+), absence by (−) and (?) indicates uncertain from microarray analyses in triplicate (see Materials and Methods). (G) Allele specific RT-PCR confirming the dynamics of imprinting for Os02g55560 in endosperm obtained from reciprocal crosses between Nip and 93-11. The RT-PCR products were cut with EcoRV. The Nip+93-11 lane is a digestion control containing equal amounts of parental Nip and 93-11 RT-PCR products.

In animals, many hundreds of imprinted genes have been identified that are thought to be involved in various functions including regulation of nutrient transfer from the foetal placenta to the embryo, in embryo growth and in adult brain development [17]–[22]. In flowering plants, disruption of imprinting alters seed development [23]–[25]. The majority of plant imprinted genes have been found in seeds of the model plant *Arabidopsis* where they manifest their biased expression in the endosperm [1]–[3]. The endosperm is a terminal seed tissue with a nutritive function that forms following double fertilization in the ovule of the flower [1]–[3], [26]. In plants, the endosperm is triploid as nuclei contain a 2 maternal∶1 paternal genome complement resulting from the fusion of the diploid maternal central cell nucleus with a sperm cell. The other product of double fertilization is a diploid zygote that develops into an embryo, the progenitor of the seedling. Embryo nuclei contain a 1 maternal∶1 paternal genome complement (Figure 1A). Until recently, only 21 imprinted genes had been identified in flowering plants from studies in eudicot *Arabidopsis* and in monocot cereals rice and maize [2]. With the exception of one maternally expressed maize embryo gene, *Mee1* [27], the remainder were imprinted in the endosperm. Transcriptomic surveys of *Arabidopsis* seeds have significantly expanded the set of imprinted genes in eudicot endosperm to over 170 candidates but additional embryo imprinted genes have not been identified [28, Wolff et al. 2011 unpublished].

In plants, nutrient allocation is considered to be the driving force for the evolution of imprinting in the endosperm according to the parental conflict theory [29]. This theory predicts that excess dosage of paternal alleles promotes larger seeds while an excess of maternal alleles produces small seeds. These predictions have held true in interploidy crosses that alter maternal and paternal gene dosage in *Arabidopsis* [23]. Some of the *Arabidopsis* imprinted genes have roles controlling endosperm cell proliferation and growth [14], [30], [31] but for most of the newly identified genes their roles in endosperm formation and nutrient allocation are unknown [28]. The recently identified *Arabidopsis* imprinted genes have a diverse range of putative functions suggesting they have the potential to influence conflict between maternal and paternal genomes at a range of molecular regulatory levels [28]. Dilkes and Comai [32] have suggested imprinting may play a wider role in plants by regulating dosage sensitive gene expression. The significance of the parental conflict theory in self-fertilizing plants like *Arabidopsis* has also been questioned and it has been proposed that imprinting may serve to promote hybridity in outcross situations [33], [34].

Different seed development profiles are observed in plants. The endosperm is transient and consumed by the embryo during seed development in *Arabidopsis*. By contrast, in rice and maize, the endosperm is persistent and contributes to the bulk of the mature seed acting as a nutrient source for the embryo at germination. Comparisons of imprinted genes found in such developmentally distinct seed types of different evolutionary origin, should provide further insight to the conservation, role and importance of imprinted genes in seed development. Prior to his study, only seven locus-specific imprinted genes had been reported in rice and maize [35]–[42]. The two known rice imprinted genes, *OsFIE1* and *OsMADS*87, were found using comparative sequence similarity with the maize imprinted gene *FIE 1* and the *Arabidopsis* imprinted gene *PHERES1*, respectively [41], [42].

We have conducted a transcriptomic analysis of imprinted genes in the embryo and endosperm of rice, an important food crop, and one in which both of these tissues persist in the mature seed. We report the identification of 262 candidate loci with parentally biased expression in the endosperm and the experimental verification of 56 of these loci. Imprinting in rice, like *Arabidopsis*, primarily occurs in the endosperm and is rare in the embryo. Only one gene was found to be maternally expressed, albeit transiently in the embryo, and it was also maternally expressed in the endosperm. A comparison of rice candidate imprinted loci with the reported set of candidate *Arabidopsis* imprinted genes indicates that these two species share very few imprinted genes in common, suggesting that imprinting is likely to have evolved independently in monocots and eudicots. Interestingly, a small number of candidate imprinted genes that share high sequence homology in rice and *Arabidopsis* are associated with epigenetic regulation, including DNA methylation, histone modification and small RNA pathways. The identified rice loci provide a comprehensive platform to further explore imprinted gene function and imprinting mechanisms in cereal seed development.

## Results

### Identification of imprinted loci in seeds from reciprocal crosses of Indica and Japonica rice

We examined imprinting in endosperm and embryos of seeds derived from reciprocal crosses between Nipponbare (Nip), a Japonica rice and 93-11, a Chinese Indica rice subspecies. The genomic sequences for these two subspecies are publically available and reciprocal crosses result in viable progeny (Figure S1). Cytological analysis of seed growth in selfed parents and in reciprocal crosses indicated that they followed previously described seed developmental profiles (Figure 1B–1D) [43]. Endosperm was nuclear in seeds 2 days after fertilization (DAF), became cellular at 3 DAF and embryo morphogenesis was complete at 10 DAF. Some variation in the timing of rice seed development was observed in the intervening stages (Figure S1A). Unlike *Arabidopsis*, rice seeds are large. They attain maximum length soon after fertilization. The embryo and endosperm are found in distinct compartments in the rice seed and they can be manually isolated as pure fractions since their boundaries are readily separable and washing of the embryo is sufficient to remove all traces of endosperm (Figure S1B). Endosperm for transcriptomic analyses was harvested at 5 DAF (Figure 1C) when seeds had nearly attained their maximum length; maternal nucellar tissue was absent and milky endosperm could readily be extracted from the top of the seed. Embryos for transcriptomic analysis were harvested at 6 DAF when the first to third leaf primordia were developing. Embryos of the same size and morphological stage were collected for RNA isolation to minimize stage variation (Figure S1B).

We conducted a genome-wide survey of gene expression in the harvested endosperm and embryo tissues using the Illumina high-throughput short read sequencing platform to identify loci that were exclusively or preferentially expressed from maternal and paternal genomes. In the endosperm, a total of 4.1 million reads could be aligned uniquely to both genomes at publicly reported SNP loci (SNP reads), covering a total of 116,291 SNPs with a median read count of 35 reads per SNP location (Table S1). In the embryo, read mapping resulted in 9.9 million reads across 168,250 SNPs and a median read count at SNP loci of 59 (Table S1). SNP reads were required to align to a unique position in both genomes covering exactly one publicly reported SNP. Throughout we considered a genomic feature expressed if it showed at least 10 uniquely aligned reads and progressed a feature for analysis if it contained at least 10 SNP reads. The use of SNP reads allows the allelic assignment of expressed regions to their parental genome of origin and the characterization of any allelic bias of expression of that region. Using this approach we were able to analyse allelic expression of approximately 50% and 70% of annotated and expressed genes across the endosperm and embryo transcriptome respectively (Table 1). Of those that we were not able to analyse approximately 20% were not annotated as containing SNPs and the remainder displayed insufficient read density across SNP loci in these datasets.

**Table 1:** Summary of imprinted candidates identified in rice embryo and endosperm by transcriptome analyses and those confirmed by PCR.

	Endosperm		Embryo	
Genes profiled with SNPs	12,313 (49.6%)		19,651 (69.7%)	
	Maternal	Paternal	Maternal	Paternal
Total imprinted loci	177	85	3^1^	0
Genes (cDNAs)	93	72	3	0
Genes with ≥90% bias	62	59	1	0
Non-genic	84	13	0	0
cDNAs confirmed	22/24^2^	27/30^2^	1/7^1^	0/0
Non-genic confirmed	6/6^3^	0/0	0/0	0/0
Loci identified with parent of origin-biased splicing	9^4^		0	

1 : Three embryo genes that met significance and four additional genes with suggestive evidence were analysed. Only one gene proved to be imprinted and it arose from the four additional genes analysed (Figure 1E). This gene is also imprinted in the endosperm.

2 : Genes with ≥90% bias were tested.

3 : Including 1 transcript from an intergenic region and five transcripts from intronic regions.

**Figure 2:**
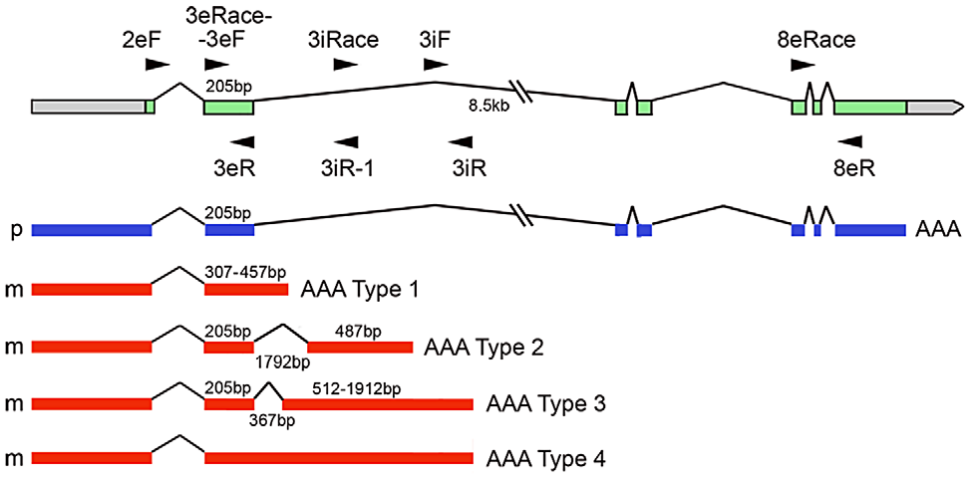
Different parentally biased transcripts arising from the Os01g70060 gene as a result of novel splicing and polyadenylation. A cartoon of the gene structure is shown with exons in green. The structure of the paternally biased transcript detected is shown with blue exons and the various maternally biased transcripts generated from the gene are shown with red exons. The positions of the primers used to detect transcripts are shown by arrows with the primer names as indicated. Further details are provided in Materials and Methods and confirmation of parental bias using RT-PCR and sequencing is provided in Figure S5. The transcripts terminate at a range of different positions as indicated in the text, and the transcripts are polyadenylated.

4 : Maternal and paternal biased transcripts of different structure were predicted to originate from these genes as a result of novel alternative splicing. One locus was confirmed to show parent of origin-biased splicing (Figure 2 and Figure S5).

We analysed allelic expression bias of both overlapping physical windows (1 kb width every 0.5 kb) tiled across the genome and publicly annotated features relevant to the transcriptome including cDNAs, introns and intergenic regions (Table S2). This redundant strategy allowed us to maximize exploration of imprinted transcripts from annotated and unannotated regions of the genome.

Physical windows were considered for analysis if they included at least 10 SNP reads in each cross. The relative allelic proportions of SNP reads within windows were observed to globally agree with the expected 1∶1 maternal to paternal allelic contributions expected in the embryo transcriptome (Figure 1A, Figure S2A and S2B). Similarly, across the endosperm transcriptome allelic expression was observed to closely approximate the expected 2∶1 maternal to paternal ratio (Figure 1A, Figure S2C and S2D). This evident discrimination in global patterns of the endosperm and embryo transcriptomes suggested that the contamination of maternal tissues in both endosperm and embryo was neglible and specific parental contributions could be determined in this dataset. Evidence for imprinting was determined through statistical analysis of observed to expected allelic contributions to expression and loci were considered putatively imprinted at *P*≤0.05 after a Pearson chi-square test.

Analysis of the embryo identified 56 windows with evidence of imprinting (55 maternal, 1 paternal) whereas in the endosperm, 1,220 windows showed evidence of imprinting (24% maternal, 76% paternal; Table S2). Allelic analysis of expression also allowed the identification of subspecies bias of expression, for example more than 2,500 windows showed increased expression of the 93-11 allele in endosperm (Table S2). These subspecies expression biases are potentially of interest for the characterisation of the genetic regulation of expression in these subspecies, however these biases were not further investigated in this study.

Imprinted physical windows showed no evidence of physical clustering throughout the genome. The median distance between significant windows was 1.1 Mb and only 4.5% (58) of all window candidates were within 10 kb of another putatively imprinted window. We observed that the majority (97%) of all significant windows mapped either to within annotated gene features or within several kilobases upstream or downstream of annotated genes. The 3% of remaining window candidate loci (41) could be classified as intergenic. They were located several kilobases away from annotated transcription, and 64% (26) of these involved significance at a single window or region of 1 kb; with the remainder involving two or at most three consecutive windows. Given the strong overlap of candidate imprinted loci with annotated features, we decided to focus our analysis on annotated features for imprinting status, however, all physical window candidate loci were investigated for overlap with imprinted candidates detected by the annotated feature analysis.

SNP reads were mapped to annotated gene features including cDNAs, introns and intergenic regions (Table S2). This mapping utilised all SNP reads analysed in the physical window analysis where reads were allocated to cDNA and intron regions as appropriate when they overlapped annotated features, and intergenic regions when they did not overlap annotated features. In addition, we considered all annotated transcript isoforms for all genes, analysing each transcript isoform independently for imprinting status. This reallocation accounted for all SNP reads analysed in the physical window analysis. Statistical analysis of the expression of annotated features identified 262 candidate loci with evidence of a parental expression bias including 177 maternally expressed and 85 paternally expressed candidate loci (Table 1, Tables S2 and S3). The majority of these candidate loci (165) coincided with annotated cDNAs, whereas the remaining loci (97) were localized to intergenic regions (75) or introns (22) (Table 1, Tables S2 and S3). These intergenic loci might represent non-coding RNAs as identified in mammals [17], [18] or various types of mis-annotated transcription. A search of miRBase (http://www.mirbase.org/) with the sequences of these intergenic loci did not detect any matches to known miRNAs. Most of the biased transcription annotated as intronic are possibly unannotated alternative splicing events as suggested by their close proximity to annotated transcripts and the observation that the flanking exon sequences show similar modes of parental bias.

In contrast to the endosperm, only three maternally expressed cDNAs were identified in the embryo transcriptome, despite the extra sequencing depth (Table 1, Tables S2 and S3). To explore whether the candidate imprinted endosperm genes are also imprinted in the embryo, expression of all 165 endosperm cDNAs showing a parental bias were examined in the embryo transcriptome (Table S3). We found that 64 cDNAs (39%) were detected in the embryo transcriptome with less than 10 SNP reads in at least one cross; thus suggesting that these genes are minimally expressed in the embryo and their expression was too low to confidently examine their imprinting status in the embryo in this dataset. Another 85 cDNAs showed no evidence of parentally biased expression including 15 which appeared to show subspecies specific expression. Only one, cDNA Os08g08960 displayed statistically significant evidence of maternally biased expression in both endosperm and embryo transcriptomes (Table S3).

A comparison of the two analytical strategies for both embryo and endosperm transcriptomes revealed that the majority of putatively imprinted physical windows overlapped with candidates suggested through the annotated features analysis. There was, however, a portion of the windows analysis (36%) not represented in the annotated features analysis, and a much smaller subset of the annotated feature candidates (7%) not captured in the physical windows analysis. In all but three cases, these analysis-specific loci overlapped annotated gene features in the rice genome. On closer inspection it was observed that some biased windows lost evidence of imprinting when summarised into larger features such as cDNA. This could suggest the presence of noise over smaller numbers of SNP loci that could be eliminated by analysis of a larger region with more SNP evidence. It could also suggest that in some cases differentially biased expression of windows within one gene may correspond to parent of origin-specific alternative splicing as found in animals [44]. As mentioned previously, our analysis of annotated features considered all annotated alternate splice variants. We extended this in a modification of the physical windows analysis to search for evidence of unannotated alternative splicing differentiated by parent of origin in the endosperm and embryo transcriptomes. This analysis identified nine candidates in the endosperm and none in the embryo that showed evidence of a novel phenomenon (Table 1 and Table S3). Multiple parentally biased transcripts of differing sequence and genic structure appeared to be arising from the same locus in the nine identified endosperm candidates as a result of alternative splicing and we termed this parent of origin-specific alternative splicing.

In summary, the collective *in silico* transcriptome analysis of the embryo and endosperm tissues of hybrid F1 seeds derived from reciprocal crosses identified 93 maternally and 72 paternally biased genes, in addition to 97 parentally biased non-genic transcripts in endosperm. The analysis also identified nine putative cases of parent of origin-specific alternate splicing. By contrast, parentally biased gene expression was restricted to only three maternally expressed genes in the embryo (Table 1). This tissue-specific profile of imprinting was also reflected in analysis of physical windows tiled across the genome which identified 20-fold more putatively imprinted loci in the endosperm relative to the embryo. The majority of putative imprinted loci identified through the window analysis were co-located with annotated transcripts either as cDNA or as non-genic regions associated with a transcript. A small subset of unannotated intergenic loci identified in both the analysis of physical windows and annotated features may be non-coding RNAs, but they do not match any currently known non-coding RNA sequences. Since the profiling utilized poly A+ RNA they are unlikely to be small RNAs. These putative intergenic transcripts will require further characterization to confirm their transcription and explore their function.

### Identified imprinted loci are not clustered

Most of the candidate rice imprinted genes and intergenic loci identified are not organized in physically co-localized clusters in the rice genome of the type found in mammals [45]. There are six pairs of candidate imprinted genes located close to each other that we term micro-clusters (Cluster 1: Os01g12860-Os01g12890; Cluster 2: Os02g29140-Os02g29150; Cluster 3: Os03g27450-Os03g27460; Cluster 4: Os05g05780-Os05g05790; Cluster 5: Os06g33640-Os06g33690; Cluster 6: Os12g32150-Os12g32170). Only one of these appears to contain a duplicated gene (Cluster 5). Whether these micro-clusters are regulated in a similar manner to mammalian clusters via an imprinting control region remains to be determined. We found no significant evidence for enrichment of transposons or repeats around the candidate imprinted rice genes relative to non-imprinted genes (based on the annotation in http://rice.plantbiology.msu.edu/; Tables S4 and S5). However, given the frequent distribution of repeats around many rice genes, and the currently less advanced annotation of transposons and repeat-like elements in the rice genome, we cannot rule out the possibility that some of these elements play a role in influencing imprinting in the rice transcriptome.

### Os10g05750 is maternally expressed in the embryo and endosperm

The three putative embryo imprinted gene candidates were further examined for maternal allele-specific gene expression in embryos by RT-PCR using gene specific primers and sequencing across SNPs (Tables S2 and S3). Imprinting was not confirmed in any of these statistically predicted candidates. We then tested four additional embryo candidates that were originally statistically excluded from analysis because they showed parentally-biased expression in one cross, but had low SNP read coverage in the other cross (Table S3). One of these, Os10g05750 was confirmed to be maternally expressed in both the embryo and endosperm (Figure 1E). Os10g05750 is a homolog of olive *Ole e 1* which encodes an allergenic protein thought to control pollen tube emergence and guidance [46]. The transcript abundance of this gene is in the mid-range of imprinted transcripts detected in the endosperm (Table S3), thus it is unlikely that it results from a major endosperm contaminant. Analysis of Os10g05750 expression in the embryo and endosperm at 6, 8 and 10 DAF showed persistence of expression of the maternal allele in the endosperm. However, biallelic expression of Os10g05750 was observed in embryos of the Nip×93-11 cross at 8 DAF and some paternal expression was observed at 10 DAF in the reciprocal cross (Figure 1E). Maternal expression of the Os10g05750 gene in the embryo during seed development is therefore transient.

### Confirmation of maternally and paternally expressed endosperm loci

The remainder of our analyses focused on candidate imprinted loci identified in the endosperm. Parent-specific gene expression biases observed in endosperm formed a continuum similar to that found for the transcriptomic analysis of imprinting in the mouse brain [18] (Table S6). For experimental confirmation, we primarily selected loci with 90% or greater expression bias from one parental allele (bias of 0.9 and above in Tables S3 and S6). Within the 165 cDNAs with parental expression bias in the endosperm, 121 imprinted genes comprising 62 maternally expressed and 59 paternally expressed gene candidates met the selection criteria of strong parental expression bias (Table S6). From this subset, we randomly selected 60 putatively imprinted endosperm loci for validation by RT-PCR sequencing across SNPs. These loci included 54 from the set of 165 expressed genes including *OsFIE1* (Os08g04290), a maternally expressed rice endosperm gene that we had previously identified [41], and six other loci showing putative imprinting within introns and intergenic regions. In sharp contrast to the single imprinted gene identified in the embryo, we confirmed a total of 22 predicted maternally expressed genes and 27 predicted paternally expressed genes in the endosperm (Table 2 and Figure S3). We also confirmed maternal expression of an intergenic region between two non-imprinted genes Os01g69110 and Os01g69120 where there is currently no annotated ORF (Table 2 and Figure S4). Os04g20774 produced transcripts with preferentially maternal reads from introns and paternal reads from coding regions and this was confirmed using exon and intron primers (Table 2; Figure S3). Maternally expressed transcription within the intron of another putatively imprinted gene, Os07g42390.1 was also confirmed (Table 2 and Figure S3). Collectively, these results provide support that the methods utilized appear robust for the identification of parent of origin-specific gene expression.

**Table 2:** Imprinted loci confirmed by RT-PCR and sequencing across SNPs in reciprocal crosses.

Bias type^1^	Gene ID^2^	Annotation	SNPs	SNP Reads	Bias	LogP	Expression^3^	Sperm expression^4^
Mat	Os01g10080.2	expressed protein	4	830	0.99	21.47	g	PPP
Mat	Os01g12890.1	expressed protein	15	310	0.96	5.54		PPP
Mat	Os01g38650.1	expressed protein	13	548	1.00	17.03		PPP
Mat	Os01g40450.1	2-aminoethanethiol dioxygenase	7	433	1.00	11.48		AAA
Mat	Os01g42270.1	transcriptional corepressor LEUNIG	9	215	1.00	5.33	en	AAP
Pat	Os01g54784.1	expressed protein	5	165	1.00	26.64	g	PPP
Pat	Os01g63250.1	josephin	4	235	0.99	38.86		PPP
Pat	Os01g70060.1^5^	unknown function, DUF618 domain containing protein	16	213	0.66	15.56	g	PAA
Mat	Os01g70060^5^	transcripts in intron	26	192	0.80	1.98	en-p	
Pat	Os02g39920.1	AT hook motif family protein	8	677	0.99	109.20	g	PPP
Pat	Os02g51540.1	aspartyl protease domain containing protein	7	286	0.99	40.33	g	PPP
Pat	Os02g51860.1	dehydration response related protein	4	77	1.00	12.30	g	PPP
Mat	Os02g55560.1	protein phosphatase 2C	4	574	0.98	14.14	en-p	AMP
Pat	Os02g57080.1	serine/threonine-protein kinase	5	177	1.00	22.53	g	PPP
Pat	Os03g27450.1	ADP-ribosylation factor	4	494	1.00	76.36	g	PPP
Mat	Os04g08570.1	PE-PGRS family protein PE_PGRS20	18	675	0.99	15.20	en-p	AAA
Pat	Os04g20774.1	pumilio-family RNA binding protein	8	211	0.99	35.95	en	PPP
Mat	Os04g20774	transcripts in intron	30	679	0.92	7.93	en	
Mat	Os04g39560.1	expressed protein	11	863	0.86	15.41	g	PPP
Pat	Os04g42250.2	transferase family protein	3	248	0.99	44.39	g	AAA
Mat	Os05g26040.1	pumilio-family RNA binding protein	21	2630	0.99	50.21	en	AAA
Mat	Os05g26040	transcripts in intron	4	520	1.00	10.18	en	
Mat	Os05g34310.1	no apical meristem protein	6	1803	0.99	40.46	en	APP
Mat	Os05g40790.1	CCR4-NOT transcription factor	11	2024	0.99	42.86	en	PPP
Pat	Os05g41220.1	SNF1-related kinase regulatory subunit beta-1	8	179	0.97	27.23		PPP
Mat	Os06g33640.1	CAPIP1, putative, expressed	9	3256	1.00	77.23	en	AAA
Mat	Os06g33640	transcripts in intron	12	1624	0.99	28.51		
Pat	Os06g40490.1	glycosyl hydrolases family 17	4	70	1.00	15.49	g	PPP
Pat	Os07g12490.1	KH domain containing protein	5	365	1.00	56.61	g	PAA
Pat	Os07g17460.1	OsFBL36 - F-box domain and LRR containing protein	9	116	1.00	22.82	en	PPP
Mat	Os07g27359.1	expressed protein	11	822	0.99	20.92	en	AAA
Mat	Os07g34620.1	expressed protein	3	633	1.00	19.58	g	AAA
Mat	Os07g42390^5^	transcripts in intron	11	259	0.97	5.14	en	
Mat	Os08g03470.1	MBTB15	7	148	1.00	3.35		PPP
Mat	Os08g04290.1	OsFIE1, WD domain, G-beta repeat domain	1	61	0.93	1.63	en	AAA
Pat	Os08g27240.1	ARID/BRIGHT DNA-binding protein	17	95	0.94	17.41	g	PPP
Mat	Os08g38850.1	phosphatidylinositol transfer	1	132	1.00	3.24	g	AAA
Pat	Os08g41710.1	FHA domain containing protein	6	284	1.00	54.86	g	PPP
Pat	Os09g03090.1	expressed protein	1	104	1.00	19.01	en-p	PPP
Mat	Os09g03500.1	ZOS9-01 - C2H2 zinc finger protein	11	581	0.99	11.89	en	
Pat	Os09g20650.1	OsFBX323 - F-box domain containing protein	2	56	1.00	9.89	g	PPP
Mat	Os09g34880.1	basic region leucine zipper domain containing	6	269	1.00	8.07	en	AAA
Mat	Os09g36470.1	retrotransposon protein unclassified, expressed	29	699	1.00	21.25		PPP
Pat	Os10g04890.1	expressed protein	9	254	1.00	51.08	en	PAP
Pat	Os10g04980.1	OsFBX365 - F-box domain containing protein	2	39	1.00	7.72		AAA
Mat	Os10g05750.1^6^	POEI3 - Pollen Ole e I allergen and extensin family	11	162	1.00	5.22	g	AAA
Pat	Os10g39780.1	protein phosphatase 2C	7	160	1.00	27.20		PPP
Pat	Os11g07910.1	transmembrane 9 superfamily member	4	76	1.00	12.85	g	AAP
Mat	Os11g27470.1	hypothetical protein	3	132	1.00	3.93	en	AAA
Pat	Os12g08780.1	YUCCA10 like, flavin monooxygenase	6	75	1.00	12.85	en	AAA
Pat	Os12g31350.1	SSXT protein	9	453	0.99	83.34	g	PPP
Pat	Os12g32170.1	hypothetical protein	4	43	1.00	9.30		
Pat	Os12g37860.1	expressed protein	14	568	0.98	81.69	g	PPP
Pat	Os12g40520.1	MATH domain containing protein	8	183	1.00	25.15	g	PPP
Mat	Os01g69110 and Os01g69120 intergenic region	7	204	1.00	1.96	en		

1 : Mat is maternal and Pat is paternal expression bias.

2 : All the loci are imprinted in endosperm and Os10g05750 is also imprinted in the embryo. The gene ID and splice isoform suffix (.1 or .2) relate to the TIGR annotation. Those lacking the isoform suffix are transcripts expressed from an intron.

3 : Expression in other plant tissues; g refers to general expression, en refers to endosperm specific expression, and en-p endosperm preferred expression where expression is found to be higher in the endosperm than in other tissues.

4 : This indicates the expression of the locus in rice sperm cells using microarray analysis. The data shown in triplicate indicates presence (P), absence (A) or marginal (M) expression in sperm cells following analyses indicated in Materials and Methods.

5 : These loci have been predicted to show novel parent of origin-biased alternative splicing. For transcripts arising from Os01g70060, see Figure 2 for summary and Figure S5 for confirmation of parentally biased transcript isoforms.

6 : This gene is imprinted in both embryo and endosperm.

### Parent of origin-biased alternative splicing and variable transcript termination result in biased parental transcripts arising from one gene

We selected Os01g70060, one of the nine candidate loci showing evidence of novel alternative splicing patterns to closely examine the types of maternal and paternal transcript isoforms generated from this gene using 3′-end race and RT-PCR. Figure 2 and Figure S5 show the range of transcript isoforms produced from Os01g70060, which encodes a putative DUF-domain containing protein. Correctly spliced paternally biased transcripts were detected with nine different polyadenylation sites within the small region downstream of the stop codon of this gene. In addition, a range of truncated, maternally biased polyadenylated transcripts were also formed. They contained the 250 bp exon and they terminated at various positions in the adjoining large intron of the gene. A total of 18 different termination sites were identified located in the 5′ end of the 2.4 kb intron. In some cases, additional alternative splicing occurred within this intron (Figure 2). There is a stop codon just inside the large intron, thus the truncated maternally biased transcripts containing the 5′ portion of the gene are likely to produce a small protein. These data provide a clear example of parent of origin-biased transcript isoforms arising from the same plant gene. The other eight candidate loci remain to be examined as does the mechanism giving rise to this phenomenon.

### Temporal expression of imprinted genes during endosperm formation and transcript presence in other plant tissues

We examined whether transcripts from the imprinted loci were seed-specific in expression or if they were also expressed in other parts of the rice plant by RT-PCR. Maternally and paternally expressed loci were clearly not all silenced in other rice tissues, nor were all maternally expressed genes restricted to the endosperm of rice. Expression analyses conducted on 46 confirmed imprinted endosperm loci (Figure S6) indicated that most paternally expressed endosperm loci were expressed in various tissues in the rice plant (17/21), while approximately half of the examined maternal loci (13/25) were specifically expressed in the endosperm at 5 DAF (Figure S6). These genes may have functions in the development of a number of plant tissues.

The imprinted genes in the endosperm were originally identified using endosperm harvested from seeds at 5 DAF. We also examined the imprinting status of 43 confirmed genes at various stages of endosperm development (3.5, 6, 8, and 10 DAF) to assess whether imprinting was persistent during seed development. In 39 examined cases biased expression was evident at 3.5 or 5 DAF and persisted in the endosperm until 10 DAF (Figure S3). In four cases (Os06g33640, Os09g03500, Os12g32170, and Os12g40520) imprinting was lost in later stages of endosperm development as expression became biallelic in at least one cross (Figure S3). We also examined the expression of these imprinted genes in isolated rice sperm cells by microarray which revealed that 37 of the maternally expressed endosperm genes examined are also expressed in sperm cells. This suggests that the paternal allele might be silenced after fertilization (Table 2 and Table S3, and Materials and Methods).

We noted that four genes (Os02g55560, Os04g39560, Os04g42250 and Os06g40490) were biallelically expressed or incompletely imprinted in the endosperm at 3.5 DAF and became uniparentally expressed at later stages of endosperm development in both crosses (Figure 1F and Figure S3). Figure 1F summarizes the data obtained for these genes showing that two become maternally expressed and two become paternally expressed (RT-PCR sequencing traces supporting these data are provided in Figure S3). We confirmed the stage specific imprinting of Os02g55560 by allele-specific RT-PCR (Figure 1G). We also subcloned and sequenced RT-PCR products from the endosperm RNAs of both crosses for Os02g55560, obtaining 21 maternal and 11 paternal SNPs (21m: 11p) in the Nip×93-11 cross and 19m: 20p paternal SNPs in the 93-11×Nip cross at 3.5 DAF. By contrast, maternal SNPs were almost exclusively detected in 5 DAF endosperm from the Nip×93-11 cross (38m: 0p) and the 93-11×Nip cross (45m: 2p). Of the two genes Os02g55560 and 0s04g39560 that become maternally biased in the endosperm at later stages of seed development, 0s04g39560 (Figure 1F) is also expressed in sperm cells (Table 2 and ), thus paternal message carry over may be a possibility at 3.5DAF [47]. Alternatively, the paternal allele may be silenced after fertilization.

In the case of the two genes that become paternally expressed in endosperm, Os06g40490 is expressed in sperm and Os04g42250 is not (Figure 1F and Table 2 and Table S3). If the latter gene is epigenetically silenced in sperm it may be activated upon fertilization or during early endosperm development. Collectively these data indicate that there are dynamic changes in imprinted gene expression during rice endosperm development. These may involve selective silencing or activation of parental alleles after fertilization during endosperm development for some genes.

### Putative functions of rice imprinted genes and their imprinted homologs in other plants

Database comparisons of the 165 candidate imprinted rice genic endosperm loci revealed that 52 (∼30%) were of unknown function. The remainder were enriched in a range of putative regulatory processes including DNA and RNA binding and signal transduction, and cellular component organization (Figure S7 and Table 2 and Table S3). For example, several rice parentally biased endosperm genes are homologous to those regulating mRNA levels in other species, including *CCR4-NOT* [48], *Pumilio* [49] and *LEUNIG* [50]. Chromatin remodelling genes, including *OsFIE1* (Os08g04290) [41] the homolog of the maize imprinted Polycomb gene *Zmfie1* [38], a putative H3 K9 methyltransferase gene (Os03g20430) and an *SSXT* type gene homologous to a component of the SWI/SNF complex [51] were also identified.

Comparisons of our 165 candidate rice imprinted loci with the known cereal imprinted loci in maize and rice [35]–[42] revealed that only the Polycomb-group genes *Zmfie1* and *OsFIE1* (Os08g04290) were in common, with both showing endosperm specific expression [38], [41]. *OsMAD*S*87* (Os03g38610) a previously reported rice imprinted homolog of *Arabidopsis PHERES 1* [42] showed no evidence of imprinting in our dataset because very low expression of *OsMAD*S*87* was observed in our hybrid endosperm with a total of only 6 reads from both reciprocal crosses.

The 165 putative rice imprinted genes found here were also compared with two recent datasets of candidate *Arabidopsis* imprinted genes identified by seed transcriptome sequencing [28, Wolff et al. 2011 unpublished] and a third dataset of *Arabidopsis* genes predicted to be imprinted by genome methylation analysis [52]. We found a total of only 27 rice candidate imprinted genes with significant homology to the three candidate *Arabidopsis* imprinted gene datasets (Table S7). Some of the imprinted genes with high sequence homology between rice and *Arabidopsis* include C3HC4 ring finger genes, *YUCCA10* potentially involved in auxin biosynthesis, rice *AUXIN RESPONSE FACTOR 18*, a *MYB* gene and protein kinases (Table S7). Nine of the genes with high homology are implicated in epigenetic regulation, potentially involved in small RNA, chromatin remodelling, and DNA methylation functions (Table 3). Examples of the latter include *ARGONAUTE* (*AGO*) and *DsRNA BINDING* (*DRB*) protein genes potentially associated with the small RNA pathway [53]. H3K9 methyltransferase genes (*SUVH*) [54] and *PICKLE*-like genes [55] are potential chromatin remodelling factors, and a paternally biased rice homolog (Os04g22240) of imprinted *Arabidopsis VARIANT IN METHYLATION 5* (*VIM5*) [28] was also detected (Table 3). Not all 27 candidate imprinted genes with high homology between rice and *Arabidopsis* have been experimentally verified. However, from current data, it is interesting to note that parental expression biases appear to be retained in 20 cases (Table 3 and Table S7).

**Table 3:** Candidate imprinted gene homologs in rice and *Arabidopsis* may have roles in epigenetic regulation.^1^^,^ 2

Rice ID	*Arabidopsis* ID	Rice Annotation
Os01g65850.1(p)^1^^,^ 3	AT4G31900(PKR2)(p)^2^	PICKLE-LIKE chromatin remodelling factor
Os05g05780.1(p)^1^	AT4G31900(PKR2)(p)^2^	protein chromatin-remodelling complex ATPase chain
Os04g22240.1(p)^1^	AT1G57800(VIM5)(p)^2^	Similar to VIM5, DNA methylation (annotated as C3HC4 ring finger)
Os05g05790.1(p)^1^	AT2G28380(DRB2)(m)^2^^,^ 4	double-stranded RNA binding motif containing protein
Os03g20430.1(m)^1^	AT1G17770(SUVH7)(p)^2^	H3 K9 methyltransferase
Os03g22900.1(m)^1^	AT4G31900(PKR2)(p)^2^	PICKLE-LIKE chromatin remodelling factor
Os02g43460.1(m)^1^	AT2G40020(m)^1^^,^ 5	similar to rmr1 in maize for paramutation
Os03g20430.1(m)^1^	AT2G24740(SUVH8)(m)^2^^,^ 5	H3 K9 methyltransferase
Os07g09020.1(m)^1^	AT1G31290(AGO3)(m)^2^^,^ 5	ARGONAUTE (annotated as retrotransposon)
Os07g09020.1(m)^1^	AT5G21150(AGO9)(m)^2^^,^ 5	ARGONAUTE
Os07g28850.1(m)^1^	AT5G21150(AGO9)(m)^2^^,^ 5	ARGONAUTE

1 : Imprinting has not been experimentally tested.

2 : Imprinting has been experimentally verified.

3 : p is paternally biased gene expression.

4 : m is maternally biased gene expression.

5 : Hypothesized to be transcripts deposited or transferred to the endosperm of *Arabidopsis* from maternal seed tissues [28].

## Discussion

### Genomic imprinting primarily occurs in rice endosperm with transcripts arising from genic and non-genic regions

Our approach to identify imprinted genes in reciprocal crosses between Nipponbare (Nip) and 93-11 Indica rice relied on the use of SNP reads to enable allelic assignment of expressed sequences to their genome of origin and the characterization of any allelic bias in expression. Using this approach we have been able to analyse allelic expression of approximately 50% and 70% of annotated and expressed genes across the endosperm and embryo transcriptome, respectively. This resulted in the identification of 262 candidate loci in the endosperm and only three in the embryo. Given that 50% of the endosperm expressed genes and 30% of the embryo expressed genes either lacked SNPs or were expressed at a level too low to be characterized, we estimate that there might be at least an additional 260 loci in the endosperm, bringing the total closer to 520 candidate imprinted loci in the rice endosperm; and potentially additional loci in the embryo if imprinting is more prevalent in earlier stages of embryo development than those examined here. By contrast, there are 750 candidate genes estimated to be imprinted in *Arabidopsis* [28].

Subsequent validation of our *in silico* data led to the confirmation of only one gene in the embryo. This *Ole e 1* homologue [46] was found to be maternally expressed in the embryo, and it was also maternally expressed in the endosperm, although imprinting of this gene was lost during later embryo development. It may be that we have missed other imprinted embryo genes because our analyses were conducted using embryos at 6 DAF. Nonetheless, we conclude that imprinting in the embryo of rice occurs at low frequency and is transient. This is supported by the observation that imprinted genes have not been found in rice seedlings [56].

By contrast, 262 loci with evidence of a parental expression bias were found in the endosperm, 177 maternally expressed and 85 paternally expressed. The majority, 165, coincided with annotated cDNAs and 97 with non-genic loci. The non-genic loci localized to introns in 22 instances, and in 75 cases to intergenic regions. Verification of the imprinting status of 92% (55/60) of candidate imprinted loci examined provided us with confidence that the method is robust, and importantly confirms the notion that in rice seed, like *Arabidopsis*, the majority of the imprinted genes are expressed in endosperm. Evidence that biased transcripts arise from intron and intergenic loci was also obtained amongst the 60 examined candidates. While the intergenic regions may be unannotated regions, it is tempting to speculate that intergenic regions encode non-coding RNAs and further characterization of this class is required.

While most of the genes examined remain persistently imprinted during rice endosperm development, we have observed dynamic changes in imprinted gene expression, including gain or loss of imprinting. Similar cases are also found in mouse, where the imprinting status of some genes depends on the stage of development [18]. Similarly, our analyses indicate that the establishment of imprinting for some rice genes may not be predetermined in gametes, and reprogramming of epigenetic marks may be effected soon after fertilization.

We found that parentally biased transcript isoforms can arise from a gene via alternative splicing and differential transcript termination. The observed transcripts are polyadenylated. Nine such putative candidate loci were identified and one investigated in detail. A similar phenomenon has also been documented at a mouse locus where the maternal allele produces functional full-length transcripts whereas the paternal transcripts are alternatively spliced in a novel manner and truncated. The production of paternally truncated transcripts was found to depend on the status of DNA methylation within the maternal allele [44]. In the case examined here in detail in rice, full-length paternally biased transcripts were formed whereas various maternally derived transcripts were truncated and the mechanisms are unknown.

In summary, we have observed that in rice seed, imprinting primarily occurs in the endosperm from both genic and non-genic regions and we have observed novel features pertaining to imprinted gene expression during endosperm development after fertilization.

### Genomic imprinting in monocot and eudicot seeds

Rice and *Arabidopsis* are developmentally distinct seed types. In rice and other cereals, the endosperm persists in the mature seed and is utilized by the embryo during germination. During cereal seed development, significant translocation of photosynthetic products from the vegetative parts of the plant occurs during endosperm differentiation and grain filling. By contrast, in *Arabidopsis*, the endosperm is transient and consumed by the developing embryo, which stores reserves for germination. Nevertheless, in both types of seeds, imprinting is predominant in the endosperm. The identified imprinted genes are not physically clustered in the genomes of these monocot and eudicot plants as found in animals, but a few paired micro-clusters are found in rice and *Arabidopsis* [Wolff et al. 2011 unpublished].

The candidate imprinted genes identified in rice and *Arabidopsis* have diverse functions which suggest that they may be involved in controlling endosperm and seed development at different regulatory levels [28]. However, only 27 of the identified 165 candidate rice imprinted genes display significant sequence homology with the candidate *Arabidopsis* imprinted genes found by transcriptome analyses [28, Wolff et al. 2011 unpublished] and predicted by methylation analyses in the *Arabidopsis* genome [52]. This contrasts with the situation observed in animals where conservation of imprinted genes and their expression modes are more frequently observed [57].

In *Arabidopsis*, imprinting appears to be a consequence of global epigenetic reprogramming during the events of sexual reproduction [52], [58]. A combination of the RETINOBLASTOMA pathway and DEMETER (DME) activity in the central cell results in a genome-wide hypomethylation of central cell DNA and the activation of maternally expressed alleles [9], [52], [58]. CG hypomethylation results from DME activity which removes methylcytosines from DNA and accompanying down-regulation of METHYLTRANSFERASE 1(MET1) and VIM5 activity. The latter functions to recruit DNA methyltransferases to hemi-methylated DNA [59], [28] The non-expressed parental allele can be silenced by DNA methylation, histone K27 tri-methylation mediated by the Polycomb group (PcG) complex, or both [7]–[14].

Genome hypomethylation has also been found in rice endosperm [60], however, a DME homologue is not evident in rice [60]. The observed hypomethylation and imprinting in rice endosperm may be due to the activity of other members of this family or by other currently unknown mechanisms [60]. Our analyses indicate that *VIM5* homologues are paternally expressed in both rice and *Arabidopsis* endosperm (Table 3). Imprinting of the putative rice *VIM5* remains to be confirmed, however, this observation suggests that some mechanisms regulating imprinting may be common in the endosperm of both species.

Analyses of *Arabidopsis* imprinted gene expression in mutant backgrounds has revealed that imprinting in a subset of maternally expressed genes is not controlled by DME, FIE (a member of the PcG complex) or MET1 suggesting additional unknown mechanisms for allele silencing and activation [28]. It has been hypothesized, but not yet proven, that these maternal transcripts may be transferred or deposited into the endosperm from surrounding maternal seed tissues. Some of the rice candidate imprinted genes have homology to this set of putative maternally deposited *Arabidopsis* genes (Table 3 and Table S7).

The low level of homology in candidate imprinted genes in rice and *Arabidopsis* suggests imprinted genes are likely to have evolved independently after the divergence of monocots and eudicots. It is possible that the development of these distinct seed types may require different sets of imprinted genes, which may contribute to reproductive isolation [34]. Testing the roles of the identified parentally biased genes in both the regulation of imprinting and in rice endosperm development will provide further insights into the molecular basis for and functional significance of imprinting in plants.

## Materials and Methods

### Reciprocal crosses in rice (*Oryza sativa*) subspecies and RNA preparation

The Japonica rice subspecies Nipponbare (Nip) and the Chinese Indica subspecies 93-11 were grown in the glasshouse at 26°C to 28°C under natural light supplemented with artificial light to achieve a 16 hour photoperiod. Emasculation of Nip florets was carried out by cutting mature florets in half to remove anthers and this was conducted early morning before anther dehiscence and exertion. A hot water treatment was used for early morning emasculation of 93-11 and the panicles were immersed in water at 43°C for 8 minutes. Reciprocal crosses between the emasculated subspecies were carried out after emasculation. Fertilization in rice occurs one hour after pollination. Nip×93-11 indicates Nip female pollinated with 93-11, and the reciprocal cross 93-11×Nip indicates 93-11 female crossed with Nip pollen. Seed development in reciprocal crosses and selfed subspecies parents was monitored cytologically and compared to previously described stages of seed development by Itoh et al. [43]. These data are presented in the text and Figure S1.

Endosperm samples from reciprocal crosses for transcriptome sequencing were harvested at 5 days after fertilization (DAF) by cutting the tip of the seed at the opposite end to that containing the embryo and gently removing a small portion of endosperm. Endosperm from 50 seeds for each cross was pooled for RNA extraction. Embryos for transcriptome sequencing were manually extracted at 6 DAF to minimize contamination with endosperm as separation from the endosperm compartment is established; they were washed three times in RNAlater and then size selected to obtain comparable developmental stages for RNA extraction. 600 embryos were harvested from each cross. RNA was isolated using the QIAGEN RNeasy Plant mini kit with *DNase* I treatment according to the manufacturer's protocol. 40 µg of total RNA from endosperm for each reciprocal cross, and 30 µg of embryo RNA for each reciprocal cross were sent to AGRF (Australian Genome Research Facility http://www.agrf.org.au), where libraries were constructed for each sample and sequenced using the Illumina platform (see below).

Additional material was subsequently harvested utilizing the above procedures for verification of imprinted genes and for developmental analyses. Endosperm samples for developmental analysis of gene expression were harvested at 3.5, 5, 6, 8 and 10 DAF, and embryos at 6, 8 and 10 DAF. Other rice tissues harvested for gene expression analyses included husk of developing seed, anther, mature ovule, root, flag leaf, stem and endosperm from both subspecies.

### Sequencing data generation

The generation of sequence libraries and sequence data was completed in accordance with recommended Illumina GAII protocols (http://www.illumina.com) by AGRF (Australian Genome Research Facility). Briefly, total RNA samples were tested for quality using the Agilent BioAnalyzer. Poly-A containing RNAs were purified and reverse transcribed to cDNAs using random primers. Double-stranded cDNAs were synthesized and these fragments were end-repaired and extended through ligation with a single ‘A’ base and then Illumina adaptor sequences. After PCR amplification, the sequencing libraries were size-selected on an agarose gel and purified for sequencing. The endosperm sequencing libraries were sequenced to a length of 36 base pairs, while the embryo library was sequenced to a length of 75 base pairs. Raw sequence data were generated by the Illumina analysis pipeline (version 1.3). For endosperm, one sequencing library was generated for each reciprocal cross. For embryos, one library was also constructed for each reciprocal cross.

### Sequence data analysis

Sequence data was pre-processed to trim off any 3′ adaptor sequence present and exclude sequences that were less than 18 bases in length or >50% repetitive in nature. A non-redundant sequence dataset consisting of unique sequences and associated total read counts for each sample was used in subsequent analyses (Table S1).

SOAP [61] was used to align the reads to the assembled Japonica (Nip) and 93-11 genomes [62], [63]. Reads were filtered to those that aligned perfectly to a single location in one genome, and to the other with exactly a single mismatch corresponding to a SNP reported in the public SNP repository (http://rice.plantbiology.msu.edu/cgi-bin/gbrowse/rice/) (Table S1). For direct comparability to the endosperm, only the first 36 bases of 75 base embryo reads were analysed. This maintained a similar risk of reads being excluded from consideration due to the presence of more than one SNP in the read. Even with this conservative approach, the embryo data were observed to have greater sequencing depth and SNP coverage than the endosperm data (Table 1 and Table S1) which is indicative of the greater yield of sequences now possible in rapidly emerging sequencing chemistries. On the basis of SNPs detected, reads were assigned to either the maternal or paternal genome in each cross. Read counts were normalised between crosses for discrepancies in total read count by conversion to reads per million and multiplication by a fixed constant to return values to integer counts similar in scale to the observed data.

Two approaches were used to complete genome-wide scans of putatively maternal or paternal reads. One approach used overlapping windows of 1 kb (every 0.5 kb) to summarise read counts across the whole genome, and the other summarised reads into annotated genic features including cDNAs, introns and intergenic regions. In both approaches only those features showing a total normalized read count of at least 10 reads for both crosses were considered (Table S2). Parental expression bias (B) of window and genic features were calculated from totals of normalised read counts across each feature. For example, maternal bias was calculated as shown below.
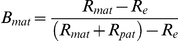
Where B_mat_ is maternal expression bias, R_mat_ and R_pat_ are the total normalized read count across the feature allocated to the maternal genome and paternal genome respectively, and R_e_ is the expected read count as calculated from proportioning the sum of R_mat_ and R_pat_ into the expected maternal∶paternal contributions (2∶1 in the endosperm; 1∶1 in the embryo) (Figure S2). Statistical significance (−log_10_ *P*) was assessed using a Pearson chi-square test (degrees of freedom = 1). Deviation from expected contributions was considered statistically significant at −log_10_ *P*≥1.3 (*P*≥0.05). Putatively imprinted features were identified as those that showed concordant statistically significant bias in both crosses associated with the gender and not the subspecies of the parent. Subspecies dependent features were identified as those that showed significant bias concordant between crosses and associated with the subspecies of the parent and not gender. Where evidence from both crosses was combined, single bias and −log_10_ *P* values were calculated as the minimum (least significant) of the two crosses.

Genome-wide scans for involvement of alternate splicing were completed in two ways. Firstly, the genic feature analysis considered all annotated alternate transcript isoforms. Secondly, a modification of the sliding window analysis allowed us to investigate the dataset for the involvement of novel, unannotated alternate splicing in transcribed regions. In both cases, the data was screened for transcripts that contained genic features or parts of genic features displaying a mixture of imprinting status. All candidates proposed by these methods were closely inspected with regard to read distribution relative to reported annotations. In cases where annotated alternate transcripts shared identical support (read counts), these were collapsed and compound annotated (Table S3). Similarly, in cases where intergenic regions were implicated and it was not possible to unambiguously determine functional association with a single neighbouring gene, these findings were collapsed and compound annotated (Table S3).

### Functional characterization of imprinted features

Putatively biased genic features in both embryo and endosperm were investigated for proximity to transposons and repeats with the rice genome and differential GC content (Tables S4 and S5). Transposon and repeat annotation were obtained from a public rice genome browser (http://rice.plantbiology.msu.edu/cgi-bin/gbrowse/rice/). At the time of download this annotation contained predictions of 264,562 repeats and transposons including MITE-like (43.7%), transposon (22.2%), retrotransposon LINE-like (0.1%), retrotransposon SINE-like (2.7%), retrotransposon unclassified (22.1%) and unclassified repetitive elements (5.4%). The analysis investigated both proximity and density of transposons and repeats to imprinted cDNAs relative to similar measures for all annotated cDNAs in the genome. Genic features were annotated with gene function ontology terms (GO terms) and GO enrichment analysis was performed using a hypergeometric test for enrichment of terms relative to expected frequencies. Expected frequencies were calculated from the annotation of all transcripts found to contain at least 10 reads in the dataset.

Identified imprinted rice candidates were investigated for sequence homology to previously reported candidates from maize [35]–[40] and *Arabidopsis* [28], [52, Wolff et al. 2011 unpublished] using blast [64]. Candidates from maize and *Arabidopsis* were aligned to the rice genome (Osa1, Release 6.1) and high-scoring (E-value<1e-10) blasts hits were ordered by increasing E-value. If any of the rice candidates were identified amongst the blast hits its highest (most significant) rank was recorded. Similarly, candidates from this study in rice were aligned to the maize (B73 RefGen_v2) and *Arabidopsis* genome (TAIR9). Consequently, the rank of 1 means that this is the best match reported in the target genome. Subsequent rank numbers can be interpreted as a measure of the number of genes (rank - 1) with better sequence homology in the target genome than the match reported. This approach not only identifies sequence homology but indicates the specificity of the homology relative to other possible sequence matches throughout the genome which is particularly relevant for comparisons in large gene families.

### Gene expression and confirmation of imprinting with RT-PCR sequencing

Primers for RT-PCR sequencing and gene expression were designed to be flanking at least one SNP and, if possible, at least one intron. About 0.5 µg total RNA from various tissues was used for cDNA synthesis using an Invitrogen Superscript III Reverse Transcriptase kit. The 20 ul PCR mix contained 2 ul of 10× buffer, 1.3 ul of MgCl_2_ (25 mM), 1 ul of dNTPs (5 mM), 0.5 ul of each primer (20 uM) and 1 unit Taq DNA polymerase (Perkin Elmer). The Thermal cycler programme includes an initial incubation of 3 minutes at 95°C, followed by 27–35 cycles, depending on the experimentally determined level of gene expression, where each cycle consisted of 20 s at 95°C, 20 s at 55°C and 1 min at 72°C, followed by 2 min at 72°C and 1 min at 25°C. PCR products were visualised on a 1.5% agarose gel with ethidium bromide. RT-PCR products generated from F1 endosperm and embryo were submitted for direct sequencing. RT-PCR products for Os02g55560 were subcloned using pGEM-T Easy Vector System (Promega) and then sequenced. Several imprinted genes were also confirmed by allelic RT-PCR.

### Confirmation of parent of origin-specific alternative splicing of Os01g70060

For a map of the gene and locations of primers utilized see Figure 2 and Figure S5. A GeneRacer Kit (Invitrogen) was initially used to examine the 3′ end of Os01g70060 transcripts from the endosperm of reciprocal crosses between Nip and 93-11. After reverse transcription using the GeneRacer Oligo dT Primer according to the manufacturer recommended protocol, primers from exon 3 (3eRace), intron 3 (3iRace) and exon 8 (8eRace) were used with GeneRacer 3′ Primer to PCR-amplify the RT products. These RT-PCR products were directly sequenced or subcloned and sequenced. Additional pairs of primers were designed from intron 3 or exons to detect detailed information regarding the parent of origin of the transcripts. These primers include a pair from intron 3 (3iF and 3iR), a pair from exon 2 and exon 3 (2eF and 3eR), and a pair from exon 3 and exon 8 (3eF and 8eR). Primers 3eF and 3iR-1 were used to detect transcripts proceeding from exon 3 into the adjacent intron 3. Note that 3eF overlaps with 3eRace. 3iRace is complementary to 3iR-1. For additional information refer to text.

### Sperm isolation, oligonucleotide microarray hybridization, and data collection

Sperm cells were isolated from mature anthers of field-grown rice (*Oryza sativa* subspecies Japonica, cultivar ‘Katy’). A centrifugation-based separation method was used for isolating sperm cells from mature pollen grains [65]. Total RNA was purified using the RNeasy plant mini kit according to manufacturer's instructions (Qiagen, http://www.qiagen.com/). For sperm cell RNA, we could not accurately determine concentration because of limited material. All accumulated isolated sperm RNA and 100 ng total RNA of seedlings and mature pollen were used for probe preparation for each of the three biological/replicates performed. Amplified sperm RNAs and pollen RNAs were used to test specificity of sperm isolation with qPCR. An 82-fold difference for *Ory s 1* (a sperm specific gene) [66] transcripts and 15 fold difference for *GCS1* (*HAP2*) (a specific sperm gene) [67] homolog transcripts between pollen and sperm cells confirmed the specificity of sperm cells.

Since the amount of starting total RNA was low (in the range of 10–100 ng per sample for sperm), the Affymetrix GeneChip Two-Cycle cDNA Synthesis Kit (Affymetrix, Santa Clara, CA, USA, http://www.affymetrix.com/) was used for target preparation with signal amplification. A mixture containing 15 µg of fragmented cRNA was hybridized to the Affymetrix 57K Rice Genome GeneChip at 45°C for 16 h. Subsequent washing and staining steps were performed on a GeneChip Fluidics Station 450 and the chips were scanned on a GeneChip Scanner 3000. The microarray data generated from all chips met quality control criteria set by Affymetrix. Materials are archived on NCBI Gene Expression Omnibus (GEO) as a data set within experimental series GSE17002. For determination of sperm expression, data was background subtracted, where background was considered to be 5% of signal and normalised using the dChip MBEI method [68]. Expression levels were averaged for the three replicates. We used the dChip protocol to determine the presence or absence calls in sperm cells (http://biosun1.harvard.edu/complab/dchip/). In this approach, *P*≤0.05 was declared present (P), marginal (M) was defined as 0.05>*P*≤0.065 and p>0.065 was considered absent (A). Expression status of imprinted genes in sperm can be seen in Table S3.

## Supporting Information

Figure S1 Seed development in rice subspecies and seeds derived from reciprocal crosses. (A) Embryo growth in seeds from selfed parents and from reciprocal crosses was examined by cytological sectioning. Comparisons of embryo morphology in seeds observed at the indicated days after fertilization provided an indication of embryo growth rate. Embryo staging was as described by Itoh et al. [43]. Embryo development was slower in both Nip and Nip×93-11 seeds, however, variability in embryo growth was observed. (B) As a result of the observations in A, embryos harvested from seeds 6 DAF were size selected prior to RNA extraction for transcriptome analyses. The figures indicate comparative morphology of the embryos collected from both reciprocal crosses. (C) Reciprocal crosses between Nip and 93-11 produce viable seed as shown.
(TIF)

Figure S2 Log_2_ normalized read counts for all 1 kb windows for the embryo (A and B) and endosperm (C and D) in reciprocal crosses. The black line denotes a 1 maternal to 1 paternal ratio, and the dashed line a 2 maternal to 1 paternal ratio. For the embryo (panels A and B), the dots representing expressed windows are distributed along the 1 maternal to 1 paternal ratio line. For the endosperm (panels C and D) the dots representing expressed windows are distributed along a 2 maternal to 1 paternal ratio line.
(TIF)

Figure S3 Verification of imprinted loci by RT-PCR and sequencing. Parental expression was investigated in the endosperm of seeds obtained from reciprocal crosses at different stages of development (3.5, 5, 6, 8 and 10 DAF). Most of the genes (39 out of 43) remained imprinted at later stages of endosperm development in at least one cross. The expression of three genes Os06g33640, Os09g03500 and Os12g32170 became bi-allelic in the endosperm of Nip×93-11 and one gene, Os12g40520 became bi-allelic in late endosperm of the 93-11×Nip cross. Figure 1F summarizes imprinting patterns for Os02g55560, Os04g39560, Os04g42250 and Os06g40490. There are 10 paternally biased genes, Os01g54784, Os01g70060, Os02g51860, Os03g27450, 0s07g12490, Os08g41710, Os09g03090, Os10g04980, Os12g32170, Os12g37860 which were not completely imprinted in the Nip×93-11 cross.
(PDF)

Figure S4 Maternally biased transcripts arising from an intergenic region between Os01g69110 and Os01g69120. (A) A genome browser screen shot of the genomic region containing Os01g69110 and Os01g69120 with the gene models indicated with green exons. The red rectangles above the gene models indicate overlapping 1 kb windows of maternally biased expression and white rectangles indicate low reads and/or or absence of parentally biased expression. The deep sequencing reads below the gene models form contigs and read density peaks corresponding to gene exons in the region, except for one contig of ∼300 bp which is an intergenic region (boxed) between the two genes. This intergenic region is associated with maternal transcripts represented by small red rectangles, and aligns with the two overlapping 1 kb windows. The genes Os01g69110 and Os01g69120 are biallelic in expression as indicated by the presence of both maternal and paternal reads associated with their exons. (B) The transcripts arising from the intergenic region in the endosperm are maternally biased as indicated by the chromatographs following RT-PCR and sequencing. The flanking genes are not imprinted. The transcripts arising from this intergenic region are endosperm specific as shown by RT-PCR in a range of plant tissue from both parents and agarose gel electrophoresis of PCR products. A PCR product is only detected in the endosperm (En) lanes present above the primer front in both parents.
(TIF)

Figure S5 Parentally biased transcripts of different structure arising from Os01g70060 (parent of origin-biased alternative splicing). (A) A genome browser screenshot of the Os01g70060 locus. The overlapping 1 kb windows at the top indicate maternally biased expression (red rectangles), paternally biased expression (blue rectangles) and/or no biased expression or low reads (white rectangles). Pink rectangles represent windows which were expressed maternally in one cross with low reads in the other cross. The gene model for Os01g70060 is shown underneath the 1 kb windows and the exons are in green. Note the orientation of the gene is 3′ to 5′ in this screen shot. Deep sequencing reads form contigs which are represented by read density peaks below the gene model and correspond to exons and also to the 5′ portion of the large intron. The SNP reads (small red rectangles) in the large intron are maternally biased and correspond to red and pink 1 kb windows. The paternally derived SNP reads (small blue rectangles) are distributed towards 3′end and correspond to blue 1 kb windows. (B) Analysis of transcripts arising from Os01g70060 in endosperm from reciprocal crosses and their parental bias using RT-PCR and sequencing. (i) The gene model for Os01g70060 has been redrawn so the gene is in the 5′ to 3′ orientation. Various pairs of primers were used to detect biallelic, maternal or paternal transcripts arising from different regions in Os01g70060. The relative positions of primers are indicated with black arrows. (ii) primers 2eF and 3eR detecting biallelic transcripts. (iii) primers 3eF and 8eR detect paternal transcripts. (iv) primers 3eF and 3iR-1 detect maternal transcripts. (v) primers 3if and 3iR detect maternally biased transcripts. Note that, in the 93-11×Nip cross a weak paternal signal was detected. A summary of the types of transcripts formed is provided in Figure 2 in the text.
(PDF)

Figure S6 Examination of transcripts from imprinted loci in other plant tissues. RT PCR was conducted to test for the presence of transcripts in various tissues of both rice subspecies. Images show agarose gels and the presence or absence of expression in husk (H), endosperm (En), anther (A), mature ovule (Ov), embryo (Em), flag leaf (Fl), stem (St) or root (R).
(PDF)

Figure S7 Enriched functional categories for identified rice imprinted genes. Genic features were annotated with gene function ontology terms (GO terms) and GO enrichment analysis was performed using a hypergeometric test for enrichment of terms relative to expected frequencies. Expected frequencies were calculated from the annotation of all transcripts found to contain at least 10 reads in the dataset. These bar charts indicate categories that were most significant. Black bars in (A) and (B) indicate frequency of biallelic genes annotated to a particular GO term within the list of all genes showing evidence of expression in the endosperm. Blue bars in (A) indicate the frequency of paternal genes annotated to a particular GO term within the list of paternally expressed genes. Red bars in (B) indicate the frequency of genes annotated to a particular GO term within the list of maternally expressed genes.
(TIF)

Table S1 Transcriptome and SNP coverage generated from the sequencing data.
(DOC)

Table S2 Transcriptome analysis approaches and summary of the analysis of parental bias.
(DOC)

Table S3 Biased genic and non-genic regions imprinted in endosperm and embryos - full description.
(XLS)

Table S4 Analysis of imprinted loci for GC content and proximity to annotated transposons and repeats.
(XLS)

Table S5 Physical distances from imprinted loci to nearest transposon.
(XLS)

Table S6 Imprinted cDNAs, introns, intergenic regions separated into different groups based on the bias values.
(XLS)

Table S7 Candidate and experimentally verified imprinted genes showing sequence homology in rice and *Arabidopsis*.
(XLS)
