# Supplementary figures and images for: A Genome-Wide Survey of Imprinted Genes in Rice Seeds Reveals Imprinting Primarily Occurs in the Endosperm
Source: PLoS Genet. 2011 Jun 23;7(6):e1002125. doi: 10.1371/journal.pgen.1002125 (PMC3121744; doi:10.1371/journal.pgen.1002125)

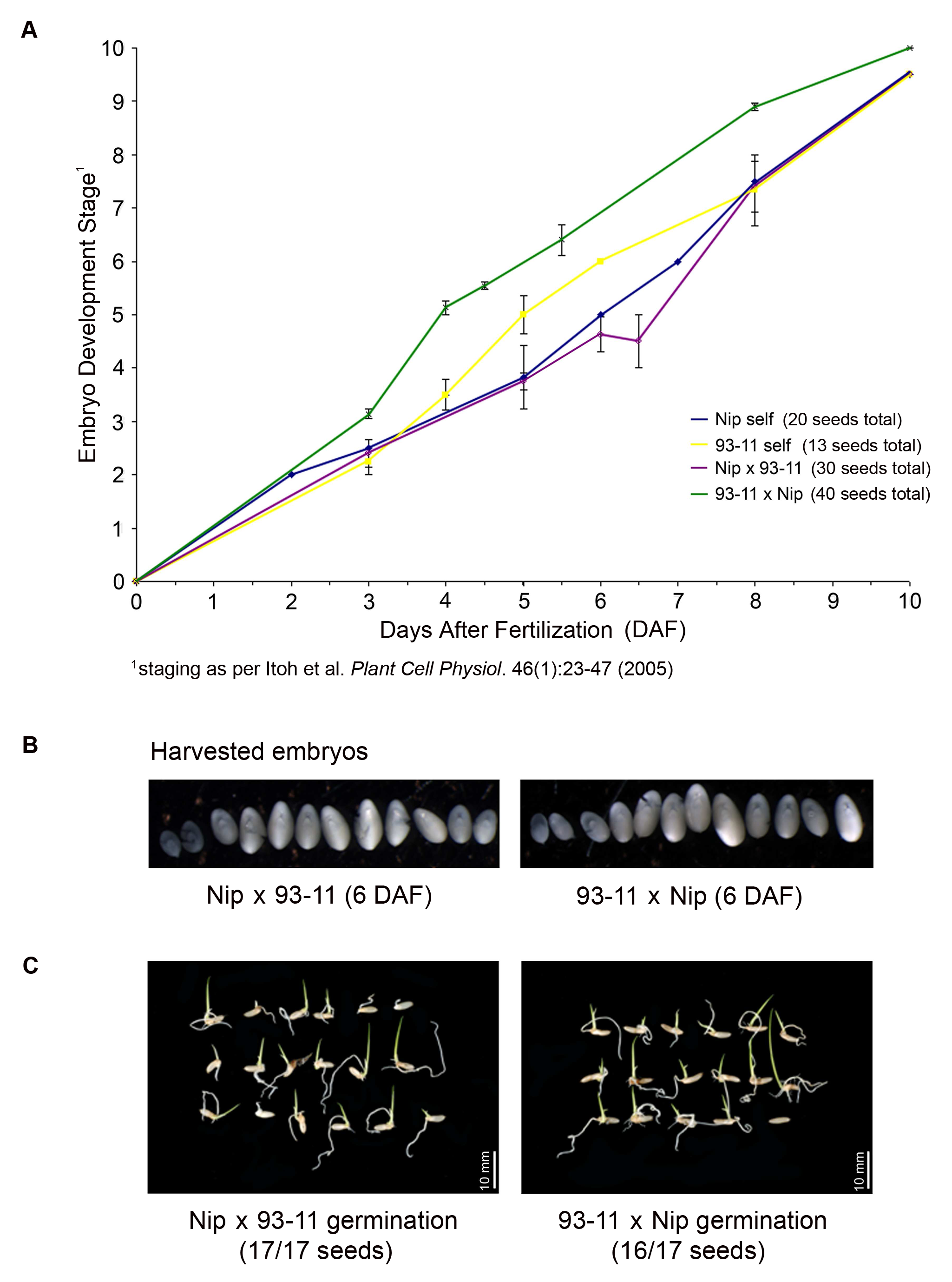

Supplement: Figure S1 — Seed development in rice subspecies and seeds derived from reciprocal crosses. (A) Embryo growth in seeds from selfed parents and from reciprocal crosses was examined by cytological sectioning. Comparisons of embryo morphology in seeds observed at the indicated days after fertilization provided an indication of embryo growth rate. Embryo staging was as described by Itoh et al. [43]. Embryo development was slower in both Nip and Nip×93-11 seeds, however, variability in embryo growth was observed. (B) As a result of the observations in A, embryos harvested from seeds 6 DAF were size selected prior to RNA extraction for transcriptome analyses. The figures indicate comparative morphology of the embryos collected from both reciprocal crosses. (C) Reciprocal crosses between Nip and 93-11 produce viable seed as shown. (TIF) [file pgen.1002125.s001.tif]

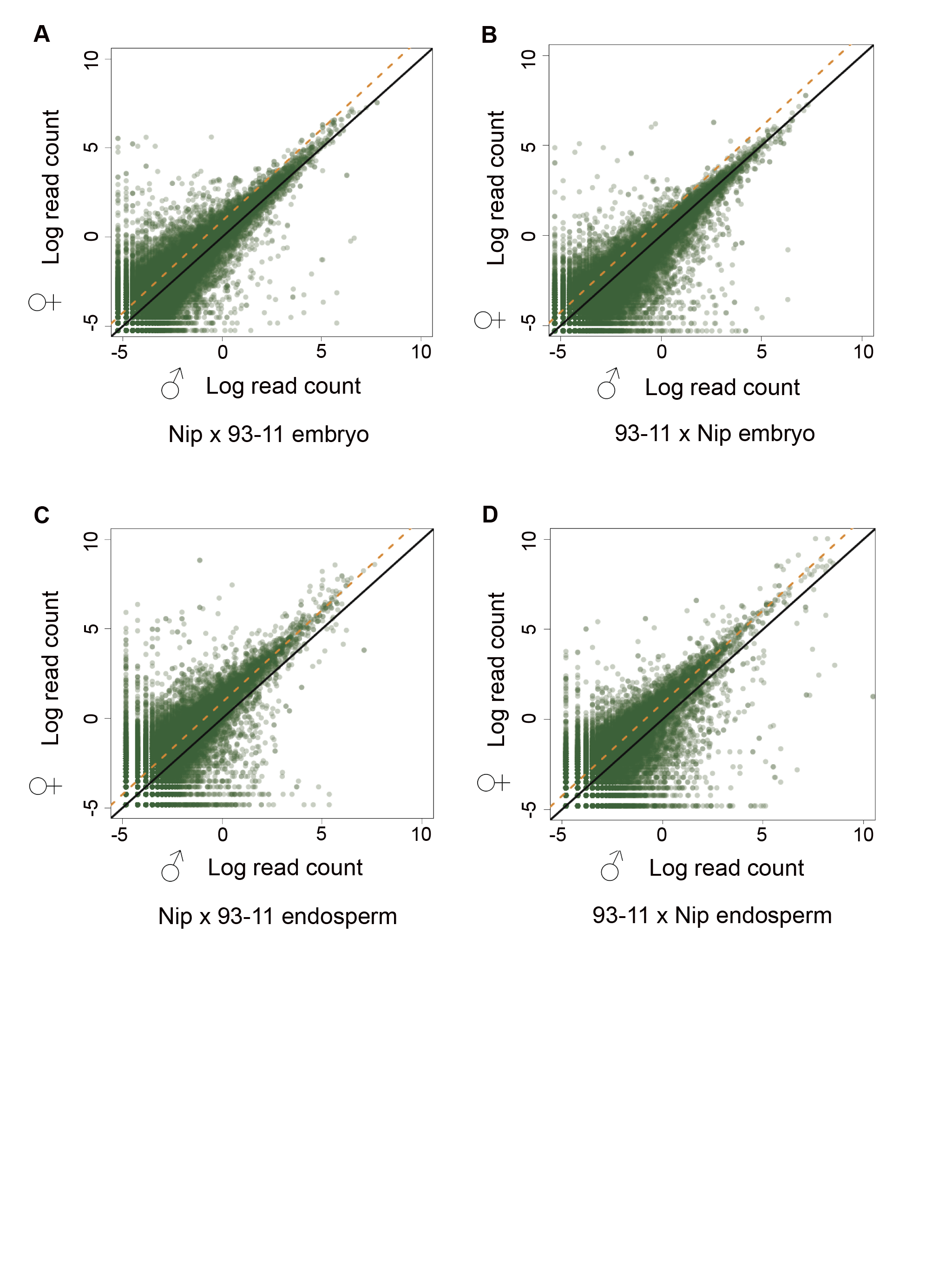

Supplement: Figure S2 — Log2 normalized read counts for all 1 kb windows for the embryo (A and B) and endosperm (C and D) in reciprocal crosses. The black line denotes a 1 maternal to 1 paternal ratio, and the dashed line a 2 maternal to 1 paternal ratio. For the embryo (panels A and B), the dots representing expressed windows are distributed along the 1 maternal to 1 paternal ratio line. For the endosperm (panels C and D) the dots representing expressed windows are distributed along a 2 maternal to 1 paternal ratio line. (TIF) [file pgen.1002125.s002.tif]

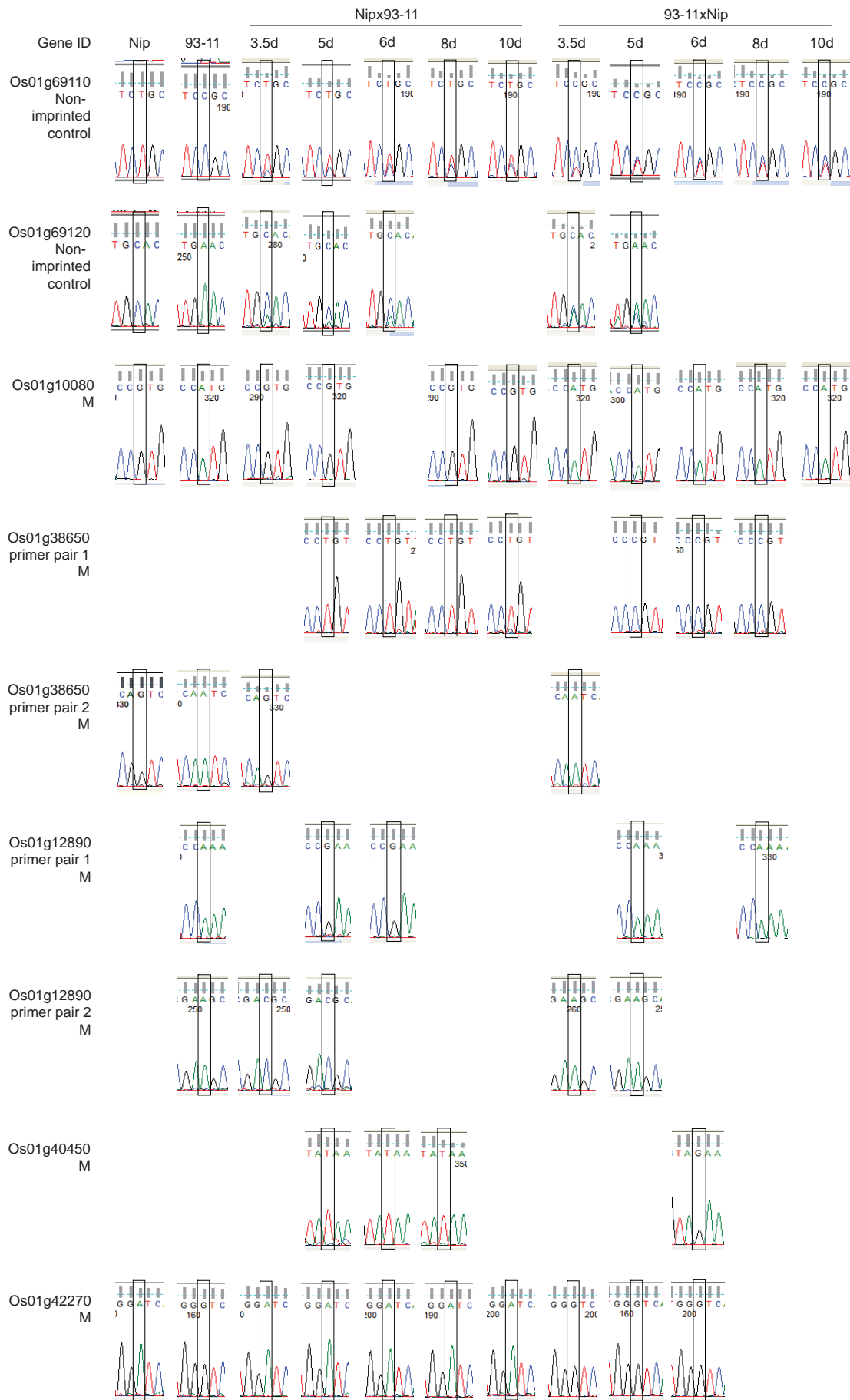

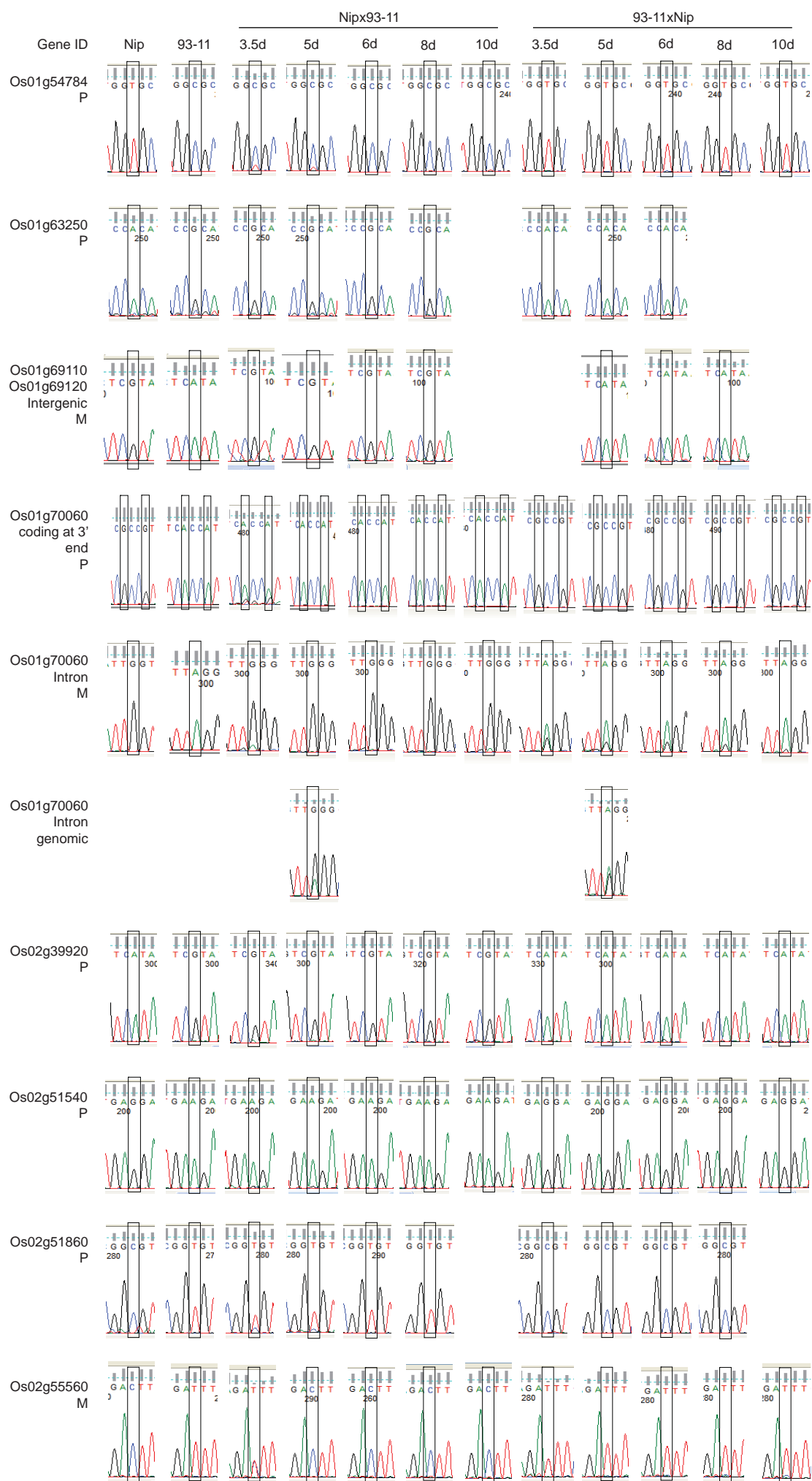

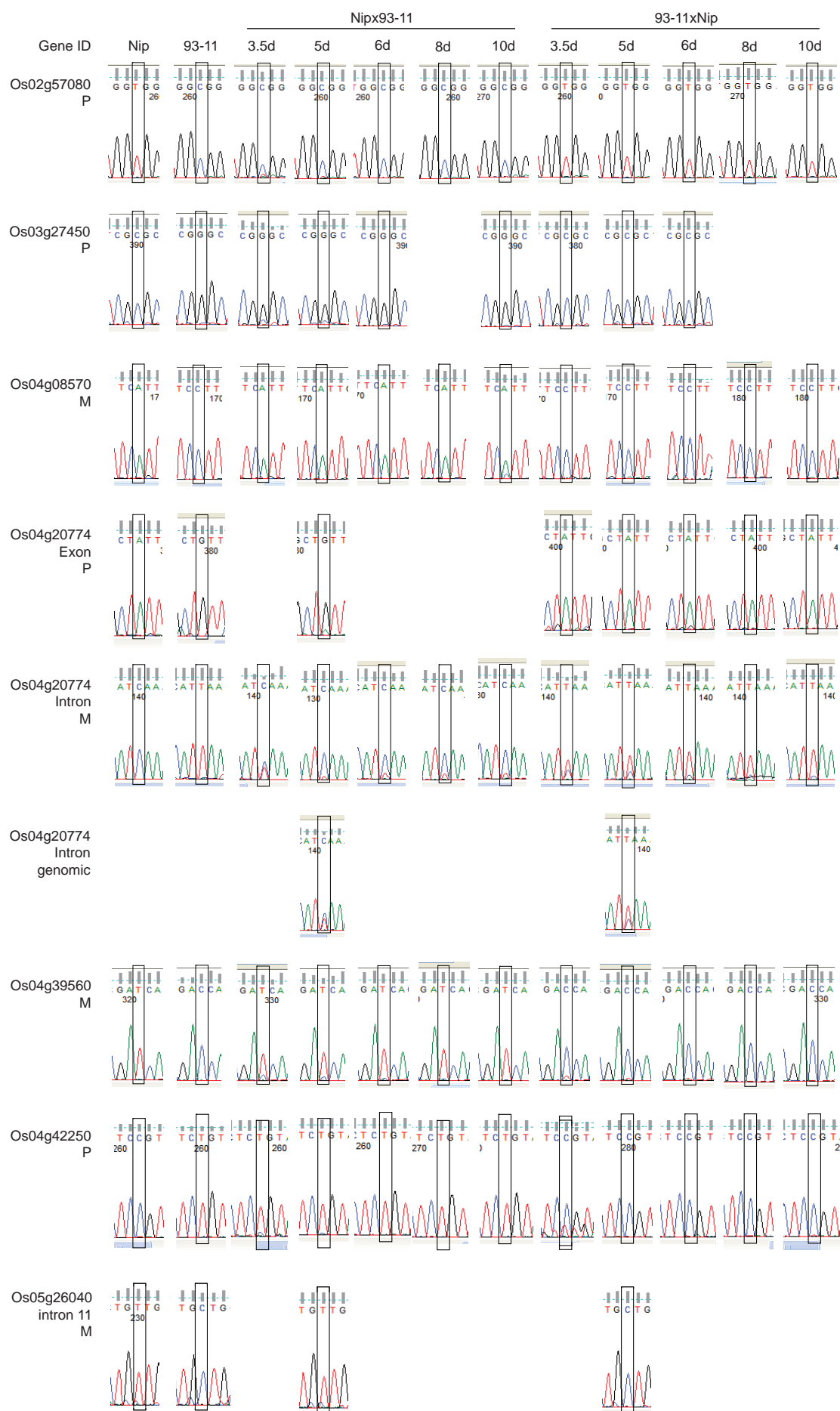

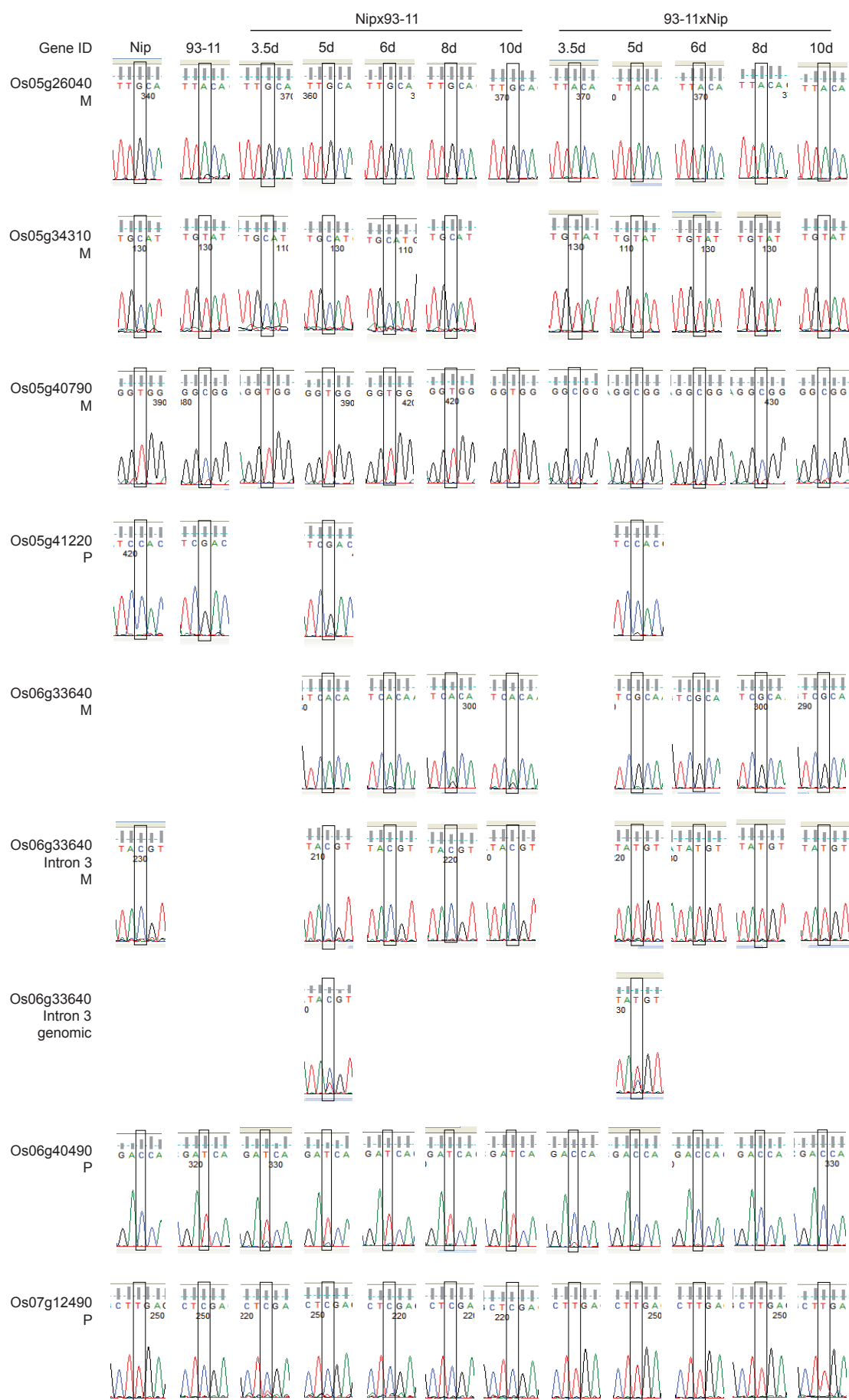

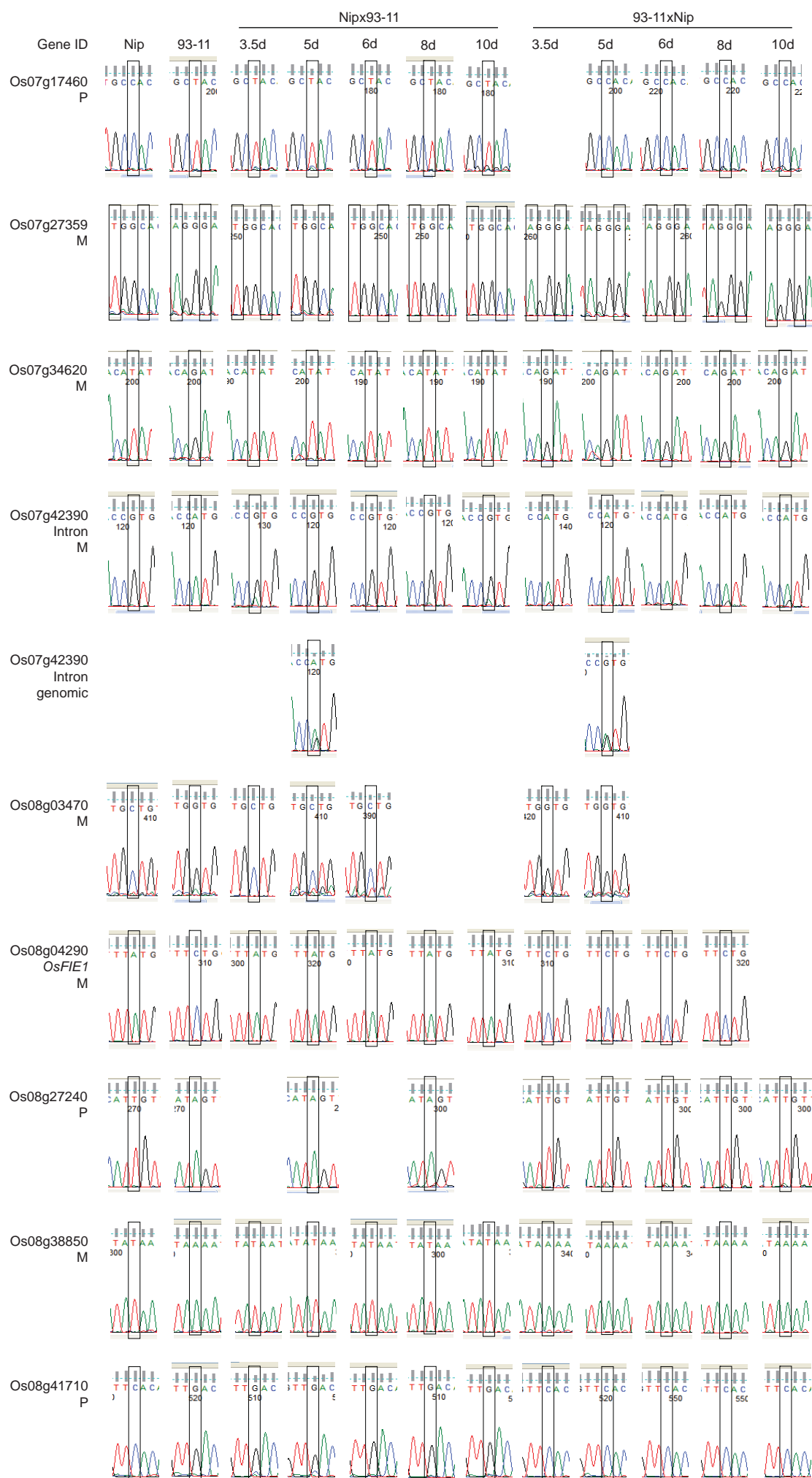

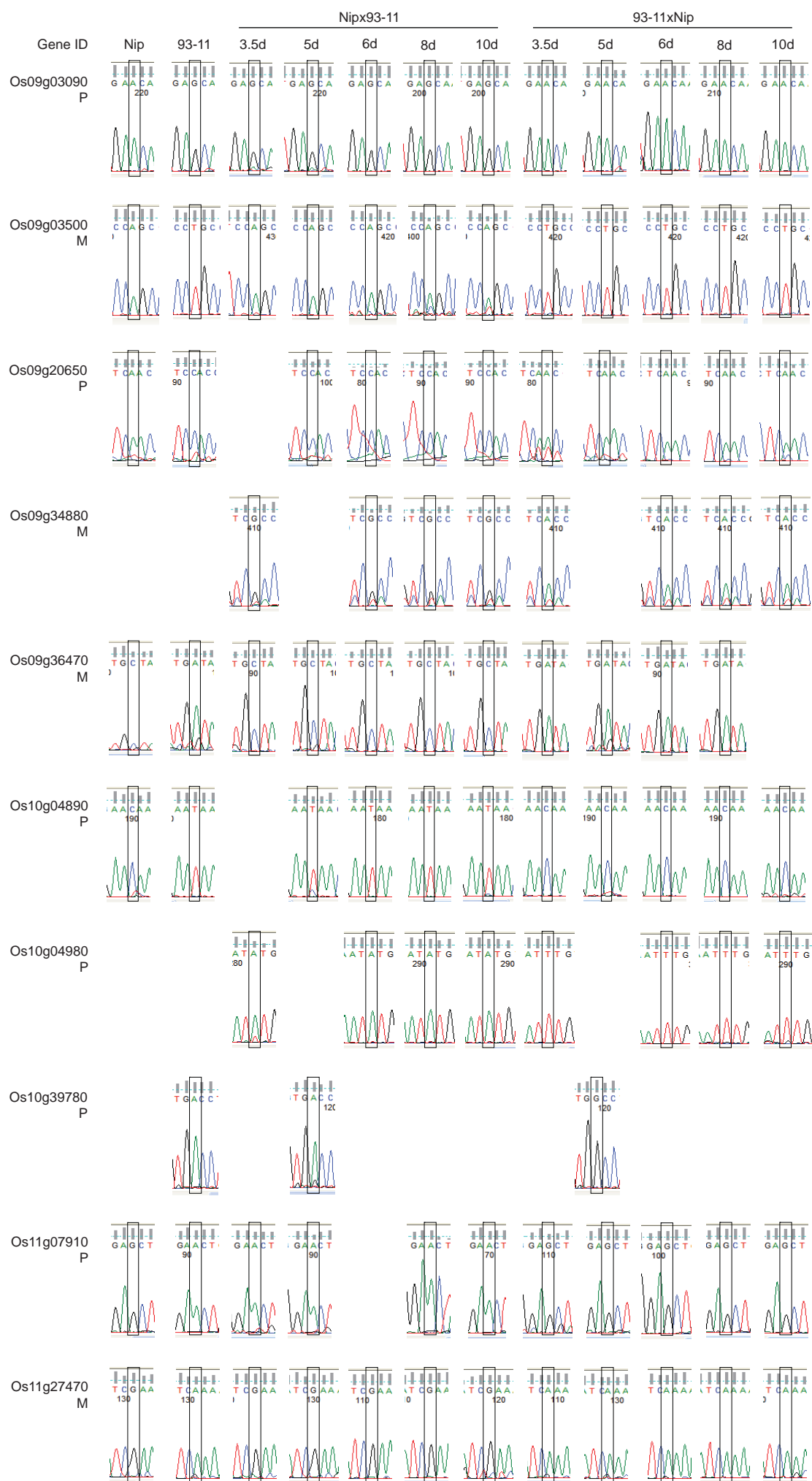

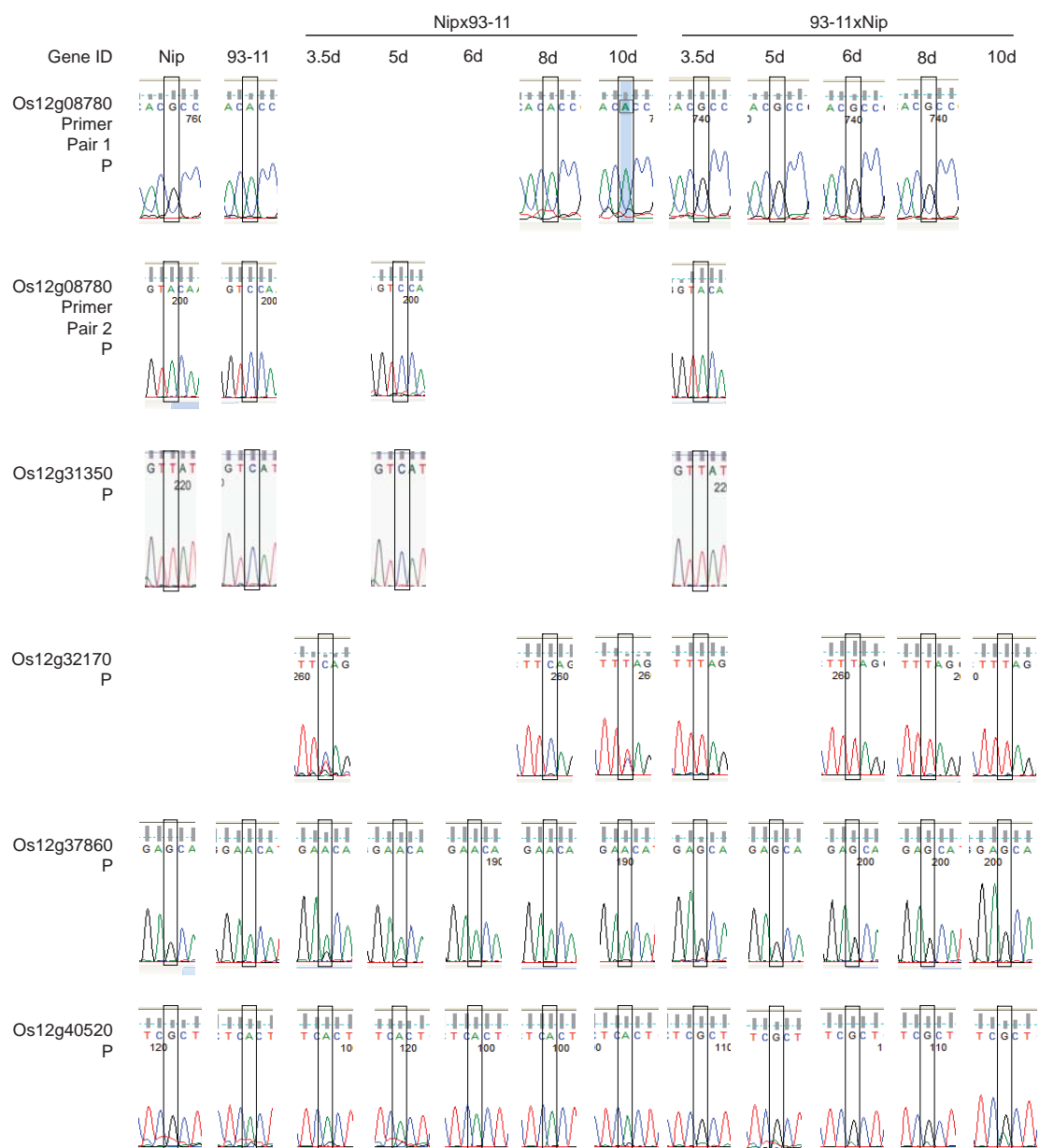

Supplement: Figure S3 — Verification of imprinted loci by RT-PCR and sequencing. Parental expression was investigated in the endosperm of seeds obtained from reciprocal crosses at different stages of development (3.5, 5, 6, 8 and 10 DAF). Most of the genes (39 out of 43) remained imprinted at later stages of endosperm development in at least one cross. The expression of three genes Os06g33640, Os09g03500 and Os12g32170 became bi-allelic in the endosperm of Nip×93-11 and one gene, Os12g40520 became bi-allelic in late endosperm of the 93-11×Nip cross. Figure 1F summarizes imprinting patterns for Os02g55560, Os04g39560, Os04g42250 and Os06g40490. There are 10 paternally biased genes, Os01g54784, Os01g70060, Os02g51860, Os03g27450, 0s07g12490, Os08g41710, Os09g03090, Os10g04980, Os12g32170, Os12g37860 which were not completely imprinted in the Nip×93-11 cross. (PDF) [file pgen.1002125.s003.pdf]

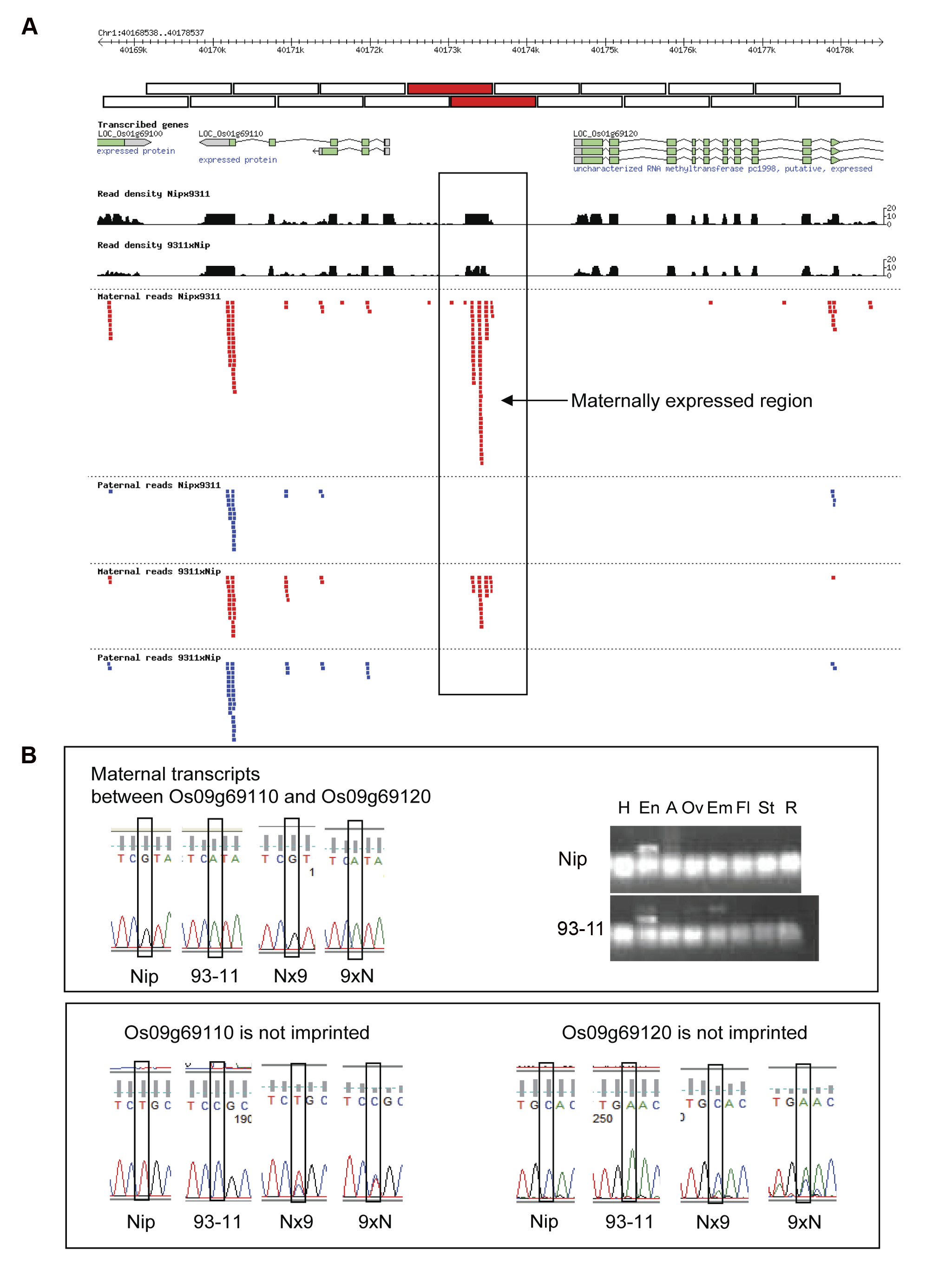

Supplement: Figure S4 — Maternally biased transcripts arising from an intergenic region between Os01g69110 and Os01g69120. (A) A genome browser screen shot of the genomic region containing Os01g69110 and Os01g69120 with the gene models indicated with green exons. The red rectangles above the gene models indicate overlapping 1 kb windows of maternally biased expression and white rectangles indicate low reads and/or or absence of parentally biased expression. The deep sequencing reads below the gene models form contigs and read density peaks corresponding to gene exons in the region, except for one contig of ∼300 bp which is an intergenic region (boxed) between the two genes. This intergenic region is associated with maternal transcripts represented by small red rectangles, and aligns with the two overlapping 1 kb windows. The genes Os01g69110 and Os01g69120 are biallelic in expression as indicated by the presence of both maternal and paternal reads associated with their exons. (B) The transcripts arising from the intergenic region in the endosperm are maternally biased as indicated by the chromatographs following RT-PCR and sequencing. The flanking genes are not imprinted. The transcripts arising from this intergenic region are endosperm specific as shown by RT-PCR in a range of plant tissue from both parents and agarose gel electrophoresis of PCR products. A PCR product is only detected in the endosperm (En) lanes present above the primer front in both parents. (TIF) [file pgen.1002125.s004.tif]

**A**

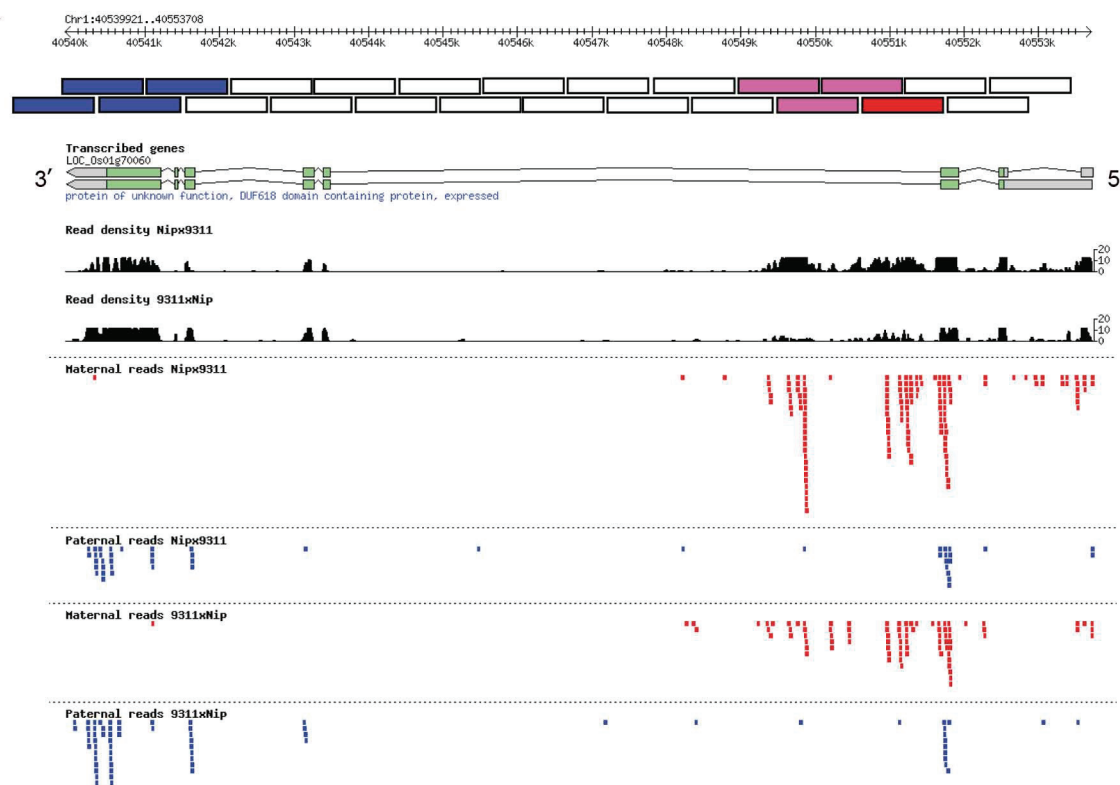

**B**

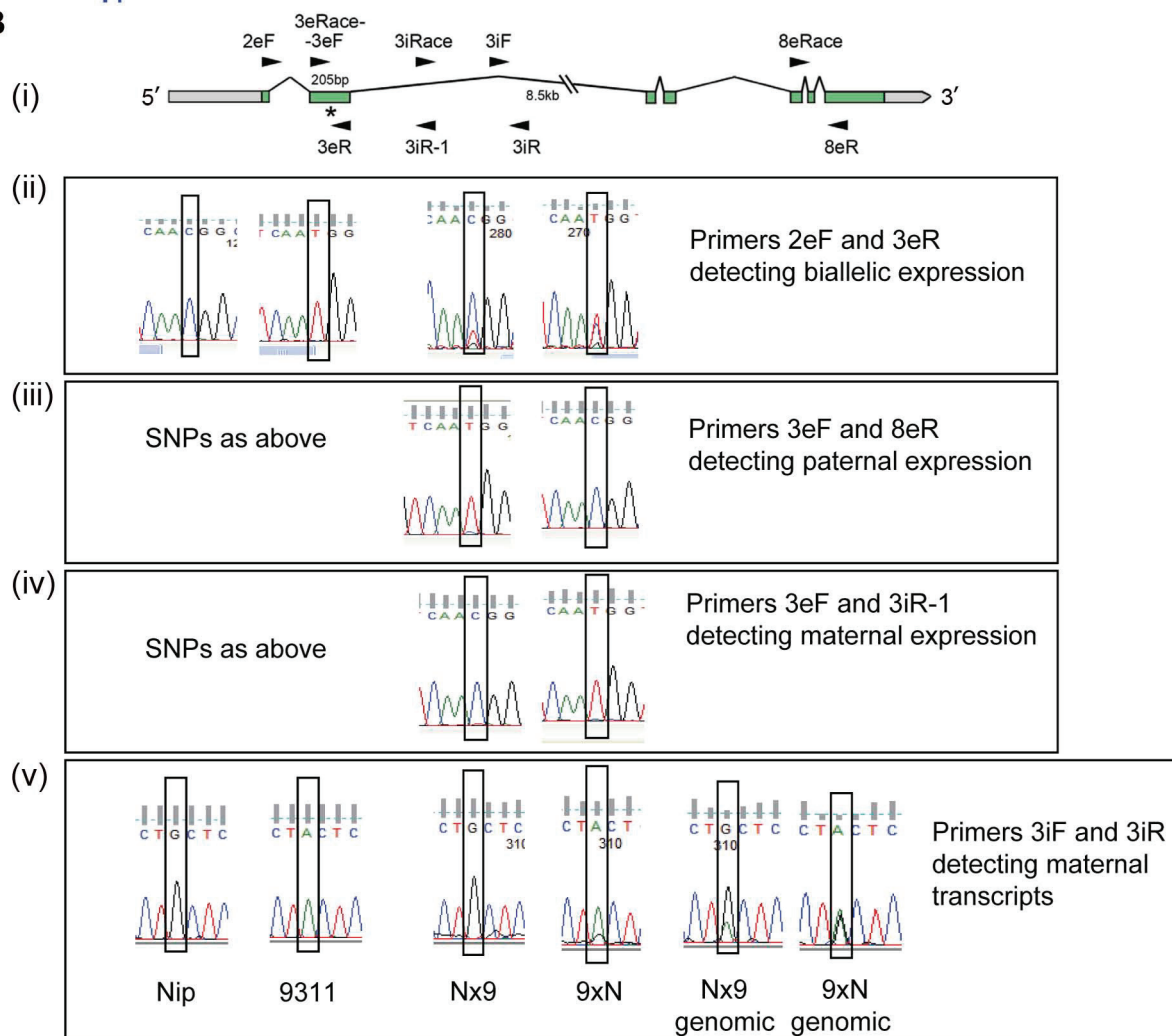

Supplement: Figure S5 — Parentally biased transcripts of different structure arising from Os01g70060 (parent of origin-biased alternative splicing). (A) A genome browser screenshot of the Os01g70060 locus. The overlapping 1 kb windows at the top indicate maternally biased expression (red rectangles), paternally biased expression (blue rectangles) and/or no biased expression or low reads (white rectangles). Pink rectangles represent windows which were expressed maternally in one cross with low reads in the other cross. The gene model for Os01g70060 is shown underneath the 1 kb windows and the exons are in green. Note the orientation of the gene is 3′ to 5′ in this screen shot. Deep sequencing reads form contigs which are represented by read density peaks below the gene model and correspond to exons and also to the 5′ portion of the large intron. The SNP reads (small red rectangles) in the large intron are maternally biased and correspond to red and pink 1 kb windows. The paternally derived SNP reads (small blue rectangles) are distributed towards 3′end and correspond to blue 1 kb windows. (B) Analysis of transcripts arising from Os01g70060 in endosperm from reciprocal crosses and their parental bias using RT-PCR and sequencing. (i) The gene model for Os01g70060 has been redrawn so the gene is in the 5′ to 3′ orientation. Various pairs of primers were used to detect biallelic, maternal or paternal transcripts arising from different regions in Os01g70060. The relative positions of primers are indicated with black arrows. (ii) primers 2eF and 3eR detecting biallelic transcripts. (iii) primers 3eF and 8eR detect paternal transcripts. (iv) primers 3eF and 3iR-1 detect maternal transcripts. (v) primers 3if and 3iR detect maternally biased transcripts. Note that, in the 93-11×Nip cross a weak paternal signal was detected. A summary of the types of transcripts formed is provided in Figure 2 in the text. (PDF) [file pgen.1002125.s005.pdf]

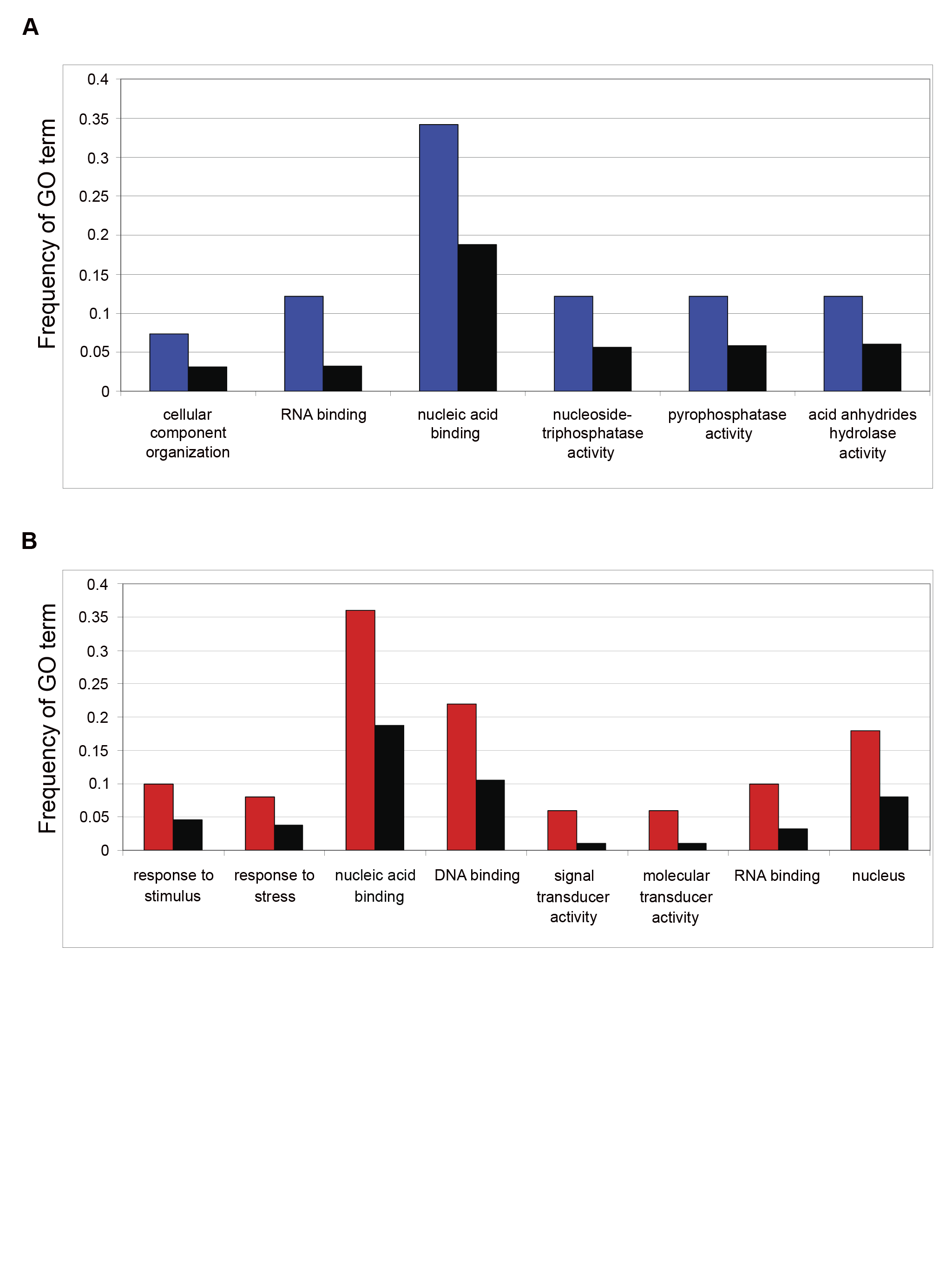

Supplement: Figure S7 — Enriched functional categories for identified rice imprinted genes. Genic features were annotated with gene function ontology terms (GO terms) and GO enrichment analysis was performed using a hypergeometric test for enrichment of terms relative to expected frequencies. Expected frequencies were calculated from the annotation of all transcripts found to contain at least 10 reads in the dataset. These bar charts indicate categories that were most significant. Black bars in (A) and (B) indicate frequency of biallelic genes annotated to a particular GO term within the list of all genes showing evidence of expression in the endosperm. Blue bars in (A) indicate the frequency of paternal genes annotated to a particular GO term within the list of paternally expressed genes. Red bars in (B) indicate the frequency of genes annotated to a particular GO term within the list of maternally expressed genes. (TIF) [file pgen.1002125.s007.tif]
