# Supplementary material for: A Genome-Wide Survey of Imprinted Genes in Rice Seeds Reveals Imprinting Primarily Occurs in the Endosperm
Source: PLoS Genet. 2011 Jun 23;7(6):e1002125. doi: 10.1371/journal.pgen.1002125 (PMC3121744; doi:10.1371/journal.pgen.1002125)
Supplement: Figure S6 — Examination of transcripts from imprinted loci in other plant tissues. RT PCR was conducted to test for the presence of transcripts in various tissues of both rice subspecies. Images show agarose gels and the presence or absence of expression in husk (H), endosperm (En), anther (A), mature ovule (Ov), embryo (Em), flag leaf (Fl), stem (St) or root (R). (PDF) [file pgen.1002125.s006.pdf]

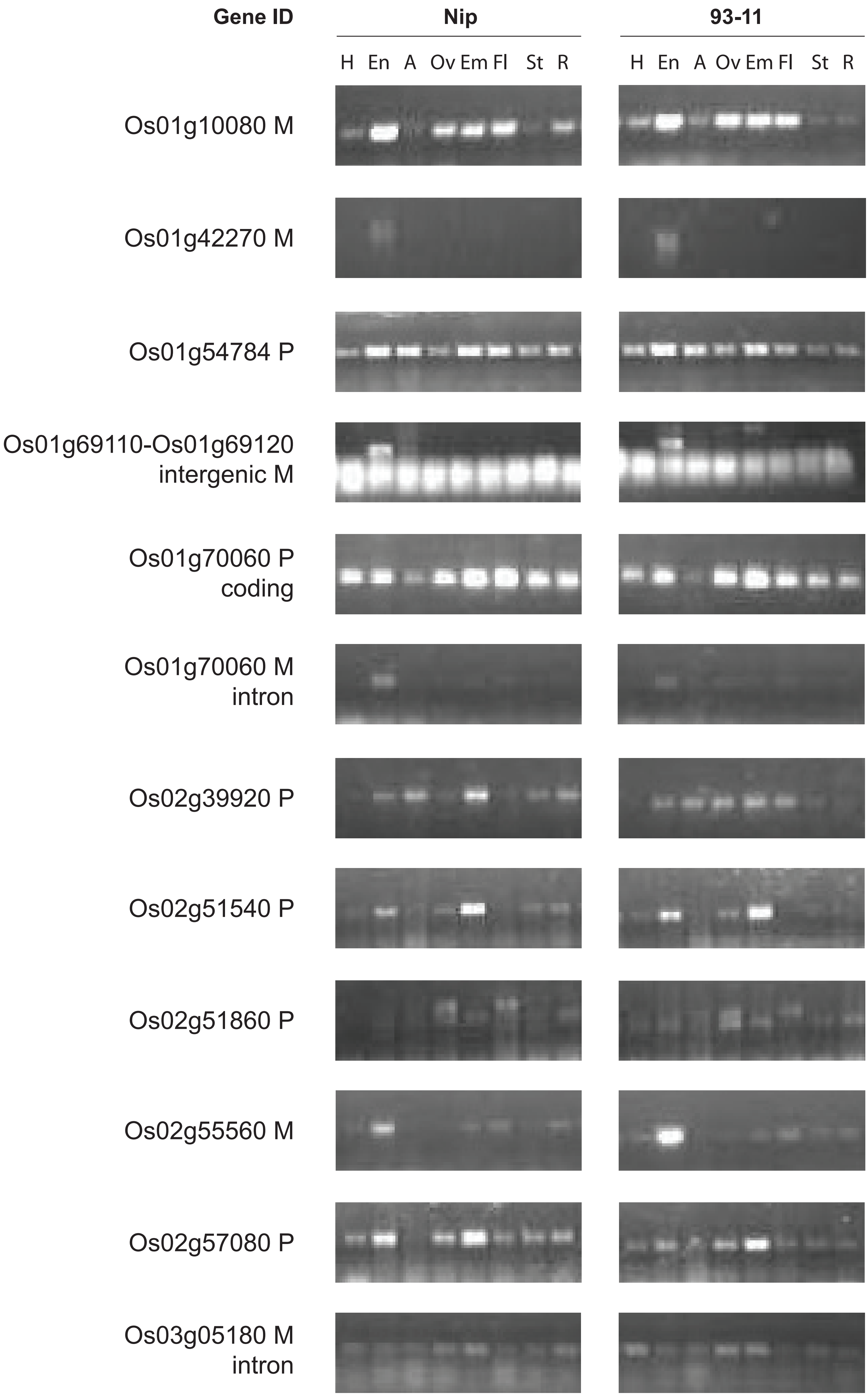
Gene ID
Nip
93-11
H En A Ov Em Fl St R
H En A Ov Em Fl St R
Os01g10080 M
Os01g42270 M
Os01g54784 P
Os01g69110-Os01g69120
intergenic M
Os01g70060 P
coding
Os01g70060 M
intron
Os02g39920 P
Os02g51540 P
Os02g51860 P
Os02g55560 M
Os02g57080 P
Os03g05180 M
intron

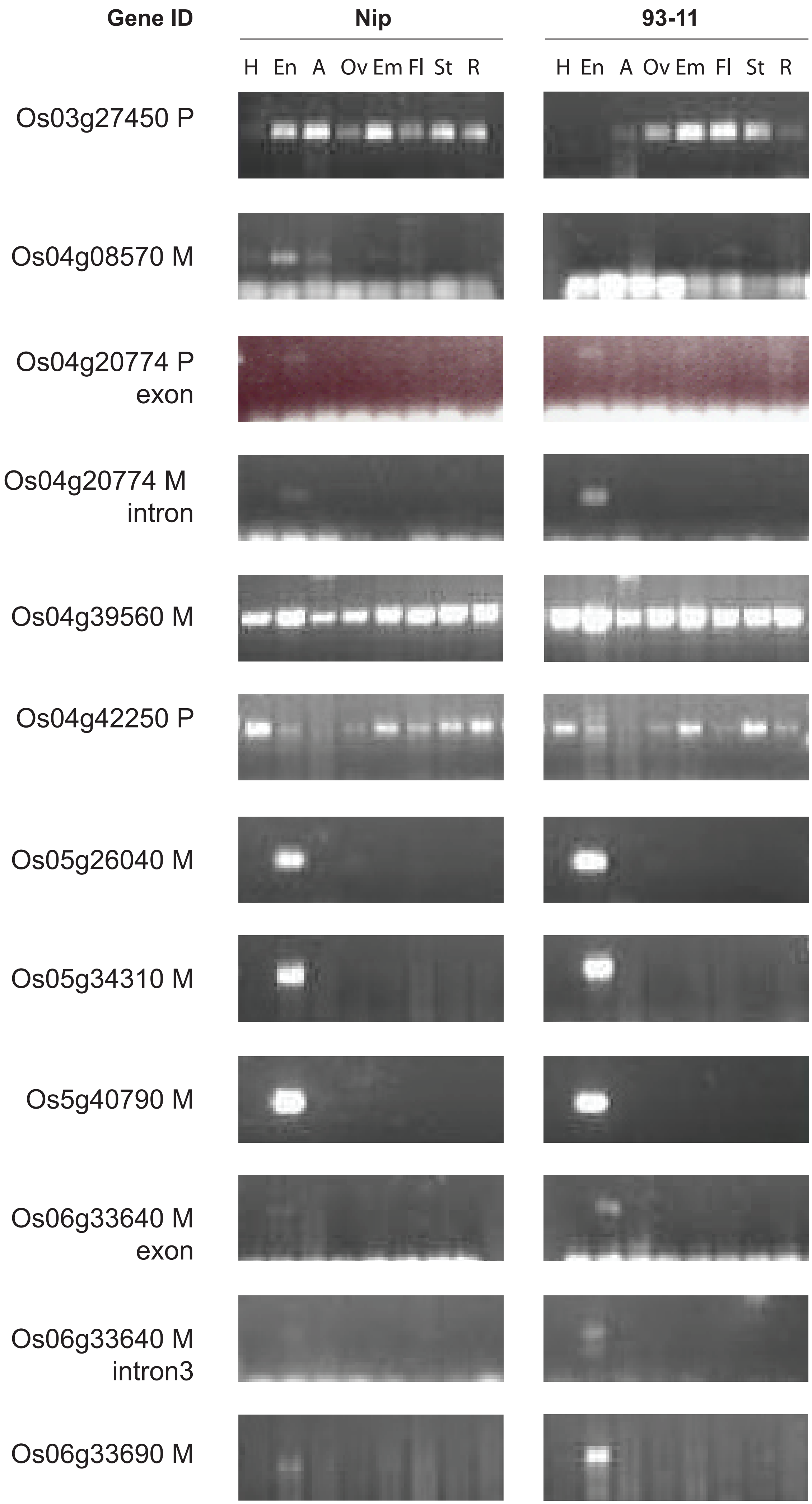
Gene ID
Nip
93-11
H En A Ov Em Fl St R
H En A Ov Em Fl St R
Os03g27450 P
Os04g08570 M
Os04g20774 P exon
Os04g20774 M intron
Os04g39560 M
Os04g42250 P
Os05g26040 M
Os05g34310 M
Os5g40790 M
Os06g33640 M exon
Os06g33640 M intron3
Os06g33690 M

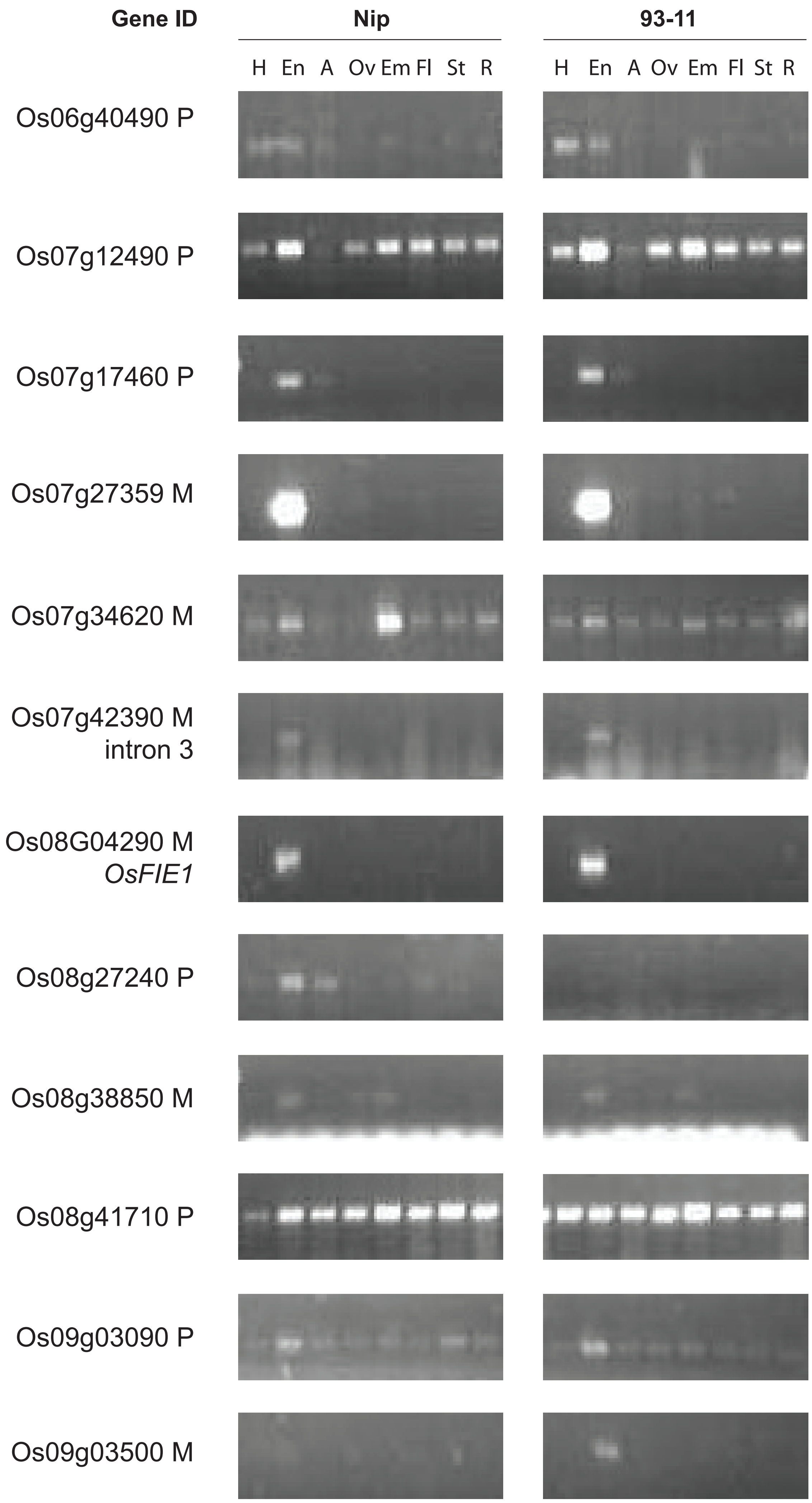
Gene ID
Nip
93-11
H En A Ov Em Fl St R
H En A Ov Em Fl St R
Os06g40490 P
Os07g12490 P
Os07g17460 P
Os07g27359 M
Os07g34620 M
Os07g42390 M
intron 3
Os08G04290 M
OsFIE1
Os08g27240 P
Os08g38850 M
Os08g41710 P
Os09g03090 P
Os09g03500 M

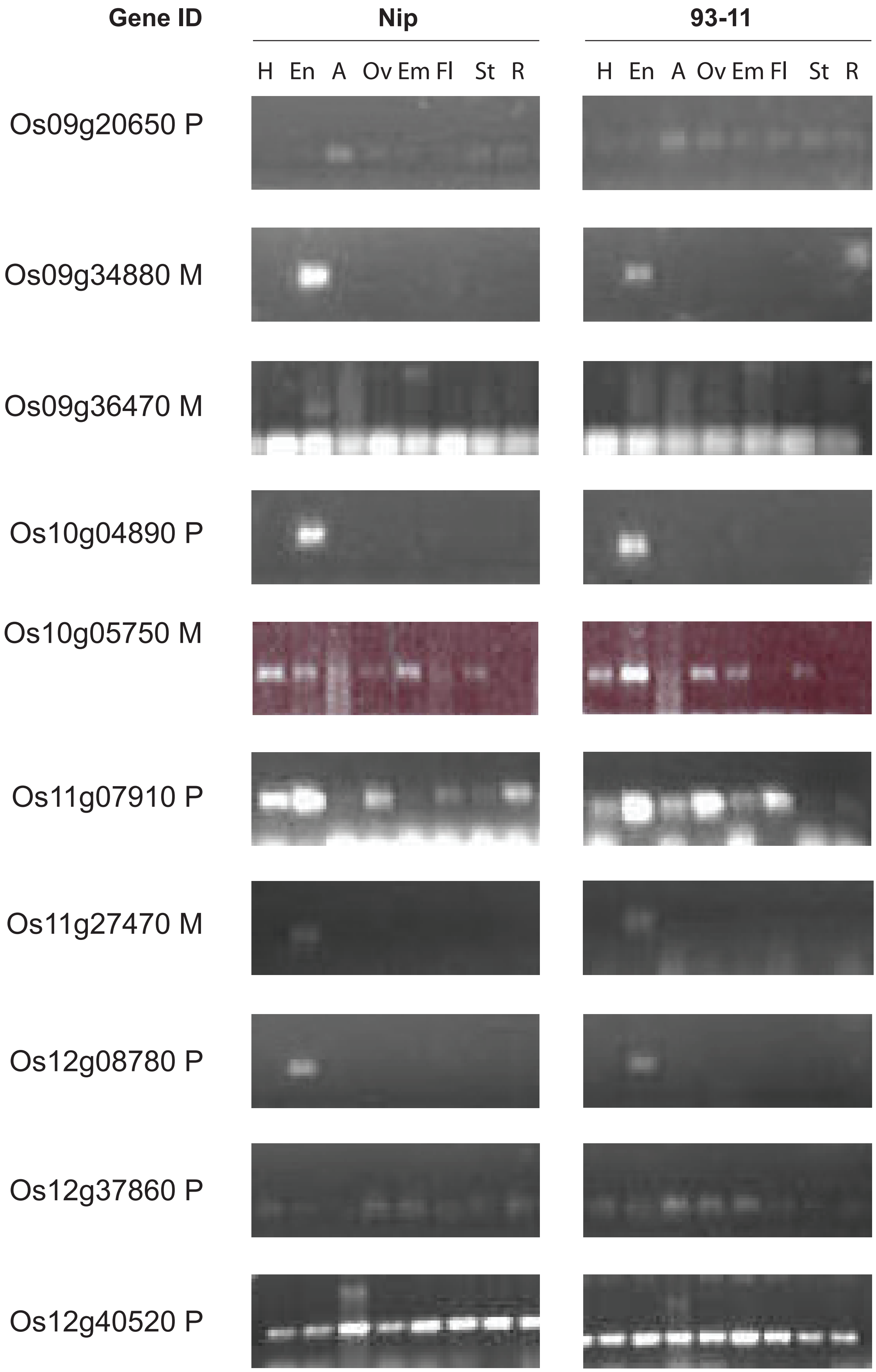
Gene ID
Nip
93-11
H En A Ov Em Fl St R
H En A Ov Em Fl St R
Os09g20650 P
Os09g34880 M
Os09g36470 M
Os10g04890 P
Os10g05750 M
Os11g07910 P
Os11g27470 M
Os12g08780 P
Os12g37860 P
Os12g40520 P
