# Supplementary material for: A Genome-Wide Survey of Imprinted Genes in Rice Seeds Reveals Imprinting Primarily Occurs in the Endosperm
Source: PLoS Genet. 2011 Jun 23;7(6):e1002125. doi: 10.1371/journal.pgen.1002125 (PMC3121744; doi:10.1371/journal.pgen.1002125)
Supplement: Table S1 — Transcriptome and SNP coverage generated from the sequencing data. (DOC) [file pgen.1002125.s008.doc]

**Table S1.** Transcriptome and SNP coverage generated from the sequencing data

|  | **Endosperm 36bp** | | **Embryo 36bp** | |
| --- | --- | --- | --- | --- |
|  | **93-11 x Nip** | **Nip x 93-11** | **93-11 x Nip** | **Nip x 93-11** |
| Total reads | 55,713,829 | 56,807,164 | 117,968,319 | 113,337,962 |
| Coverage of TS^1^ | 20.45 fold | 20.85 fold | 43.32 fold | 41.59 fold |
| Public SNPs | 2,169,320 | | 2,169,320 | |
| SNPs in TS | 347,553 | | 347,553 | |
| No. SNPs / kb of TS | 3.54 | | 3.54 | |
| SNP reads^2^ | 4,133,063 | | 9,958,616 | |
| SNPs detected^3^ | 116,291 | | 168,250 | |
| Median reads / SNP | 35.54 | | 59.19 | |
| SNPs detected in TS | 71,310 | | 90,014 | |

1: Based on <http://rice.plantbiology.msu.edu/riceInfo/info.shtml>, the transcript (TS) length (total exons) is 98066 kb.

2: Reads mapping to public SNPs

3: SNPs detected in at least one read
