# Supplementary material for: A Genome-Wide Survey of Imprinted Genes in Rice Seeds Reveals Imprinting Primarily Occurs in the Endosperm
Source: PLoS Genet. 2011 Jun 23;7(6):e1002125. doi: 10.1371/journal.pgen.1002125 (PMC3121744; doi:10.1371/journal.pgen.1002125)
Supplement: Table S2 — Transcriptome analysis approaches and summary of the analysis of parental bias. (DOC) [file pgen.1002125.s009.doc]

**Table S2.** Transcriptome analysis approaches and summary of the analysis of parental bias

|  | **Endosperm** | | **Embryo** | |
| --- | --- | --- | --- | --- |
|  | **93-11 x Nip** | **Nip x 93-11** | **93-11 x Nip** | **Nip x 93-11** |
| **Physical Windows** |  |  |  |  |
| With reads^1^ | 246,092 | 259,929 | 283,049 | 282,225 |
| With SNP reads^2^ | 30,783 | 38,610 | 64,736 | 63,738 |
| Total (nr)^3^ | 28,167 | | 59,746 | |
| Parental Bias^4^ | 294 (m) ; 926 (p) | | 55 (m) ; 1 (p) | |
| Subspecies Bias | 2,598 (93-11) ; 631 (Nip) | | 6,545 (93-11) ; 2,473 (Nip) | |
|  |  |  |  |  |
| **Annotated Features** |  |  |  |  |
| With reads (≥10) | 310,748 | 337,614 | 398,319 | 401,192 |
| With SNP reads | 17,273 | 20,638 | 31,694 | 32,621 |
| Total (nr) | 15,932 | | 29,720 | |
| Parental Bias | 177 (m) ; 85 (p) | | 3 (m) ; 0 (p) | |
| Subspecies Bias | 1,695 (93-11) ; 236 (Nip) | | 3,502 (93-11) ; 1,144 (Nip) | |
|  |  |  |  |  |
| **Genes (cDNAs)** |  |  |  |  |
| With reads (≥10) | 27,511 | 28,887 | 30,758 | 31,024 |
| With SNP reads | 12,835 | 15,032 | 20,220 | 20,337 |
| Total (nr) cDNAs with reads | 24,802 | | 28,210 | |
| Total (nr) cDNAs with SNP reads | 12,313 | | 19,651 | |
| Proportion of Genes Covered | 49.6% | | 69.7% | |
| Parental Bias | 93 (m) ; 72 (p) | | 3 (m) ; 0 (p) | |
| Subspecies Bias | 923 (93-11) ; 107 (Nip) | | 1,592 (93-11) ; 467 (Nip) | |

1: With at least 10 reads total per feature in each cross

2: With at least 10 SNP reads (reads aligning to publically annotated SNPs) in each cross

3: Non-redundant (nr)

4: Maternally biased loci (m); Paternally biased loci (p)
